# Effects of the Treatment with Flavonoids on Metabolic Syndrome Components in Humans: A Systematic Review Focusing on Mechanisms of Action

**DOI:** 10.3390/ijms23158344

**Published:** 2022-07-28

**Authors:** Henrique J. C. B. Gouveia, Mercedes V. Urquiza-Martínez, Raul Manhães-de-Castro, Bárbara J. R. Costa-de-Santana, José Pérez Villarreal, Rosalío Mercado-Camargo, Luz Torner, Jailane de Souza Aquino, Ana E. Toscano, Omar Guzmán-Quevedo

**Affiliations:** 1 Postgraduate Program in Nutrition, Health Sciences Center, Federal University of Pernambuco, Recife 50670-901, PE, Brazil; henriquegouveia.93@hotmail.com; 2 Studies in Nutrition and Phenotypic Plasticity Unit, Department of Nutrition, Federal University of Pernambuco, Recife 50670-901, PE, Brazil; raulmanhaesdecastro@yahoo.com.br (R.M.-d.-C.); barbara.jrcs@gmail.com (B.J.R.C.-d.-S.); aeltoscano@yahoo.com.br (A.E.T.); 3 Laboratory of Neuronutrition and Food Engineering, Instituto Tecnológico Superior de Tacámbaro, Tacámbaro 61650, Michoacán, Mexico; lnmurquiza@live.com; 4 Centro de Investigación Biomédica de Michoacán, Instituto Mexicano del Seguro Social, Morelia 58330, Michoacán, Mexico; luz_torner@yahoo.com; 5 Department of Nutrition, Federal University of Pernambuco, Recife 50670-901, PE, Brazil; 6 Postgraduate Program in Neuropsychiatry and Behavioral Sciences, Federal University of Pernambuco, Recife 50740-600, PE, Brazil; 7 Instituto Tecnológico Superior de Uruapan, Uruapan 60015, Michoacán, Mexico; jose.pv@uruapan.tecnm.mx; 8 Facultad de Químico Farmacobiología, Universidad Michoacana de San Nicolás de Hidalgo, Morelia 58000, Michoacán, Mexico; ros421@hotmail.com; 9 Laboratory of Experimental Nutrition, Department of Nutrition, Federal University of Paraiba, João Pessoa 58051-900, PB, Brazil; jailane.aquino@academico.ufpb.br; 10 Department of Nursing, CAV, Federal University of Pernambuco, Recife 55608-680, PE, Brazil

**Keywords:** biomarkers, metabolic syndrome, polyphenols, dyslipidemias, lipid metabolism, insulin resistance, blood pressure, inflammation

## Abstract

Diets high in bioactive compounds, such as polyphenols, have been used to mitigate metabolic syndrome (MetS). Polyphenols are a large group of naturally occurring bioactive compounds, classified into two main classes: non-flavonoids and flavonoids. Flavonoids are distributed in foods, such as fruits, vegetables, tea, red wine, and cocoa. Studies have already demonstrated the benefits of flavonoids on the cardiovascular and nervous systems, as well as cancer cells. The present review summarizes the results of clinical studies that evaluated the effects of flavonoids on the components of the MetS and associated complications when offered as supplements over the long term. The results show that flavonoids can significantly modulate several metabolic parameters, such as lipid profile, blood pressure, and blood glucose. Only theaflavin and catechin were unable to affect metabolic parameters. Moreover, only body weight and body mass index were unaltered. Thus, the evidence presented in this systematic review offers bases in support of a flavonoid supplementation, held for at least 3 weeks, as a strategy to improve several metabolic parameters and, consequently, reduce the risk of diseases associated with MetS. This fact becomes stronger due to the rare side effects reported with flavonoids.

## 1. Introduction

Metabolic syndrome (MetS) is a condition characterized by metabolic changes that are associated with an increased risk of developing cardiovascular disease (CVD) and diabetes [1,2]. Its prevalence in the world is around 20 to 25% in the adult population and depends on several parameters, such as age, sex, ethnicity, and socioeconomic status [3,4,5]. A joint interim statement published in 2009 by several associations, including the National Heart, Lung, and Blood Institute, the American Heart Association, and the World Heart Federation, introduced the criteria needed to define MetS [2]. Rather than focusing on just one criterion, they defined that individuals must have changes in at least three of the five risk factors, which include: abdominal obesity (waist circumference of ≥102 cm for men and ≥88 cm for women), elevated triglycerides (TG) (≥150 mg/dL), reduced high-density lipoprotein cholesterol (HDL-c) (<40 mg/dL in men <50 mg/dL in women or treated for dyslipidemia), elevated blood pressure (≥130 mmHg systolic blood pressure or ≥85 mmHg diastolic blood pressure or treated for hypertension), and elevated fasting glucose (≥100 mg/dL or treated for hyperglycemia) [2].

**Figure 1:**
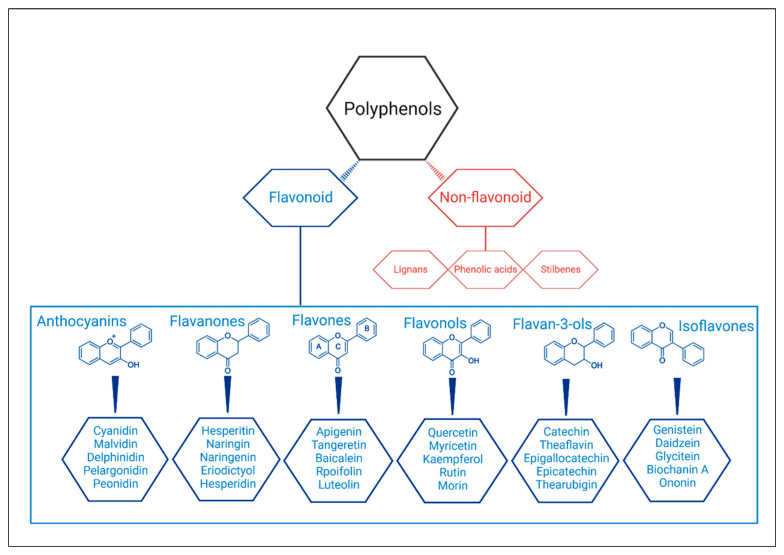
Structures and classification of the polyphenols and the main flavonoids.

In fact, abdominal obesity is the main factor of the MetS because it is associated with a complex neuroendocrine alteration [6]. The comorbidities associated to MetS are also the main death causes around the world according to World Health Organization statements [7]. Therefore, developing strategies to treat them are a major concern to scientific community. In addition to MetS drug treatment, physical activity, and dietary modifications remain the best alternatives for treatment [4]. A recent review published by Paley and Johnson (2018) stated that ‘exercise is a medicine in its own right’ and that evidence points to reduction in abdominal obesity after regular and consistent practice [8]. Diets high in bioactive compounds, such as Mediterranean diet, have also been used successfully to treat MetS [9]. This type of diet is rich in polyphenols, suggested as a promising agent in the management of MetS [10,11]. Polyphenols are a large group of bioactive plant compounds (over 8000) and have two main classes called non-flavonoids and flavonoids [4,12]. The non-flavonoids compounds include phenolic acids, stilbenes, and lignans, and the flavonoids compounds include flavonols, anthocyanidins, anthocyanins, isoflavones, flavones, flavanols (or cathechins), flavanones, and flavanonols [13] (Figure 1).

The flavonoids are widely distributed in foods, such as fruits (especially citrus), vegetables, tea, red wine, cocoa, and grains. They are responsible to provide flavor and color [14]. Increasing interest in studying these compounds has grown due their potential health benefits in most of the diseases with high prevalence in the world, such as diabetes, hypertension, and cancer. Several studies have shown the benefits of these substances on the cardiovascular system, on the nervous system, and on cancer cells, and it is believed that this is due to their antioxidative, anti-inflammatory, anti-mutagenic, and anti-carcinogenic properties, in addition to the modulation of enzymes functions [14,15,16,17]. Epidemiological studies already link increased consumption of flavonoid-rich foods with reduced cardiovascular morbidity and mortality [18,19]. Although there are reviews evaluating the effects of such compounds on the metabolic syndrome, the studies evaluated the effects of polyphenols in general or a specific type, such as resveratrol, and generally included foods, extracts, and supplements [4,20]. Thus, the present review aimed to specifically evaluate the effects of flavonoids on the components of the MetS in humans when offered exclusively as supplements over the long term, in addition to tracing the mechanisms of effects for each flavonoid subtype.

## 2. Methods

### 2.1. Systematic Review Reporting and Protocol Registration

This systematic review complies with preferred report items for systematic reviews and meta-analysis (PRISMA) [21]. Our review was carried out using a protocol published the international prospective register of systematic reviews (PROSPERO) database (registration number CRD42022304507).

### 2.2. Search Strategy

The literature search was conducted from January to February 2022 in the following databases: Medline/PubMed (1966–2022), SCOPUS (1969–2022), EMBASE (1947–2022), and Web of Science (1900–2022). It was carried out in electronic databases by two independent reviewers (Gouveia H. J. C. B. and Urquiza-Martínez M. V.) based on a predefined protocol. The search strategy was composed of terms relating to describing the intervention and outcomes. The guiding question was composed of the points of PICO, which consist of population: humans reproducing any of the MetS signs and symptoms; intervention: treatment with flavonoids; comparison: a control group treated with placebo; and outcomes: reported results on abdominal obesity (waist circumference (WC)), body weight (BW), body mass index (BMI), levels of blood glucose (BG), total cholesterol (TC), high-density lipoprotein cholesterol (HDL-c), low-density lipoprotein cholesterol (LDL-c), triglycerides (TG), blood pressure, and/or insulin resistance (IR). The primary outcomes were randomized controlled clinical trials using pure flavonoids with a control group, which evaluated metabolic outcomes, such as BW, BMI, WC, systolic blood pressure (SBP), diastolic blood pressure (DBP), LDL-c, HDL-c, TG, TC, BG, blood insulin, and IR. The secondary outcomes comprised metabolic parameters associated with oxidative/antioxidant status and chronic inflammation.

Combination of medical subject headings (MeSH) descriptors and keywords were included in the search: flavonoids (MeSH) OR flavones (MeSH) OR flavonols (MeSH) OR isoflavones (MeSH) OR flavanones (MeSH) OR anthocyanins (MeSH) OR favanols (non-MeSH) AND metabolic syndrome (MeSH) OR biomarkers (MeSH) OR metabolic parameters (non-MeSH) OR body weight (MeSH) OR waist circumference (MeSH) OR cholesterol (MeSH) OR triglycerides (MeSH) OR systolic blood pressure (non-MeSH) OR HDL (non-MeSH) OR abdominal obesity (non-MeSH). Database-specific filters for clinical trial or randomized controlled trial were used. A third reviewer (Costa-de-Santana, B. J. R.) was consulted, when needed as a mediator, for the definition of inclusion or exclusion of items when there was no agreement between reviewers. 

### 2.3. Inclusion Criteria 

To select the articles for this systematic review, the screening of studies was performed in two phases. In phase 1, screening of studies was performed by reading the title and abstract of original papers. Then, in phase 2, the full texts of the studies were considered. The following inclusion criteria were applied: randomized controlled clinical trials with a pure flavonoid intervention, which reported results related to MetS. Additionally, the following exclusion criteria were adopted: articles not found in full version; those articles that use exclusively animals or in vitro techniques; food or beverages directly as the source of flavonoids or co-intervention with other substances; studies with subjects who had an illness, such as diabetes, non-alcoholic fatty liver disease, cancer, arthritis; articles that did not report results of our interest. For search and selection of articles, there was no year of publication restrictions, as well as no restriction of the article’s language. 

### 2.4. Data Extraction

Data extraction was performed by two researchers using a piloted form. The following details were extracted from each study: article name, authors, year of publication, and journal of publication. The flavonoid (any of the variations) present in the treatment was extracted. The characteristics of subjects used in the studies were extracted: range of age, health status, and gender. The following information regarding the characteristics of the intervention was addressed: type of flavonoid; route of administration, dose/concentration used, and duration of treatment. Finally, we analyzed the effects of the treatment with flavonoids on BW, BMI, abdominal obesity (WC), blood pressure, and metabolic parameters (BG, insulin and IR, LDL-c, HDL-c, TC, and TG).

### 2.5. Analysis of Risk of Bias

The risk of bias was carried out using the Cochrane ROB tool for human studies, one for cluster randomized trials (parallel groups) and the other for crossover trials (individually randomized) [22]. These tools evaluate five domains: randomization process; deviations from the intended interventions (for parallel groups) or bias arising from period and carryover effects (for crossover trials); missing outcome data; measurement of the outcome; and selection of the reported result (mostly for parallel groups) [22]. We synthesized the information in summary tables, in a figure and in a descriptive summary, enabling comparison of results. The Review Manager Software Package Version 5.4.1 (The Cochrane Collaboration, London, UK, 2020) was used to create figures of risk bias summary.

## 3. Results

### 3.1. Search Results 

**Figure 2:**
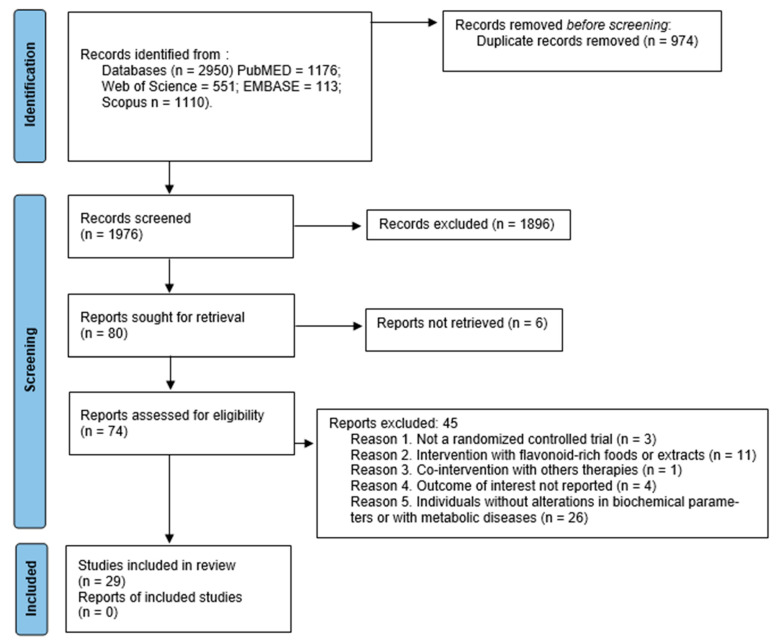
PRISMA flow diagram of study selection process. From: [23].

After a first search with the combinations of descriptors selected, 2950 articles were identified through an electronic database. After the exclusion of the 974 duplicate articles, and the analysis of the 1976 titles and abstracts, 74 articles were remaining to a full assessment for eligibility. Finally, 45 articles were excluded, and 29 articles were included in the final analysis. All the studies were published in English. Figure 2 shows the flowchart of the research that shows how this selection was performed.

### 3.2. Assessment of Quality of Studies

**Figure 3:**
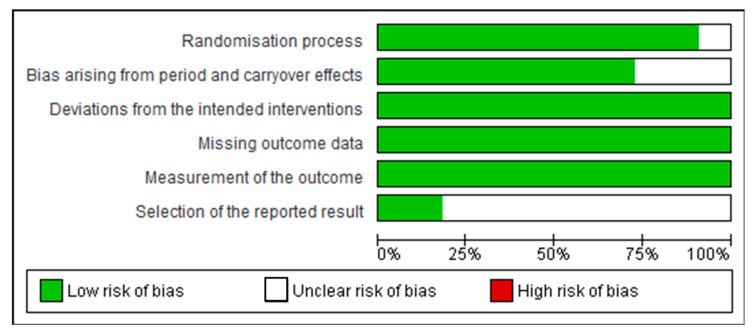
Risk of bias graph: review authors’ judgements about each risk of bias item presented as percentages across all included cluster randomized trials (parallel groups).

**Figure 4:**
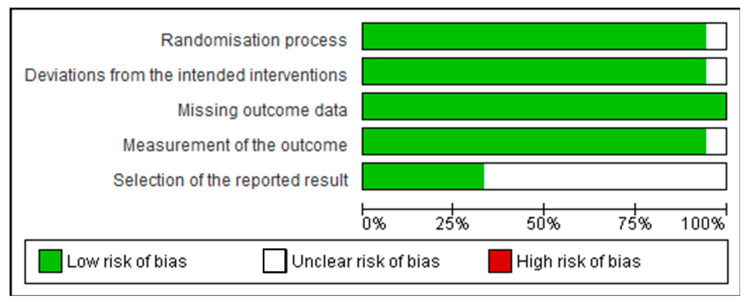
Risk of bias graph: review authors’ judgements about each risk of bias item presented as percentages across all included crossover trials (individually randomized).

The risk of bias was carried out using the Cochrane ROB tool for human studies, one for cluster randomized trials (parallel groups) and the other for crossover trials (individually randomized). All of them had random sequence allocation and no baseline differences that could suggest a problem with the randomization process. Except for one study [24], all the other studies were double-blind, and only two crossover studies did not report whether there was sufficient time for any carryover effects to have disappeared before outcome assessment in the second period [25,26]. All studies compared data from treated subjects with placebo data for all outcomes. In general, these studies did not show biases that warrant further discussion (Figure 3 and Figure 4).

### 3.3. Clinical and Methodological Characteristics 

All studies are randomized controlled trials, however, eighteen are randomized trials (parallel groups) and the other eleven are crossover trials (individually randomized). The studies evaluated subjects with dyslipidemia or pre-dyslipidemia [27,28,29,30,31], hypercholesterolemia or hypertriglyceridemia [25,32,33,34,35], prehypertension [36,37,38,39,40], prediabetes [24,41], being overweight or obese [42,43,44,45,46,47], and metabolic syndrome [26,44,48,49,50,51] (Table 1 and Table 2). 

### 3.4. Intervention

Eleven studies have been conducted with anthocyanin. In these studies, the dose used ranged from 40 to 640 mg per day, and the duration ranged from 2 to 24 weeks [24,25,27,28,29,32,34,36,37,52,53]. Six studies used hesperidin. The dosage ranged from 146 to 1000 mg per day, lasting from 3 to 12 weeks [35,38,42,48,49,50]. Four studies were conducted with quercetin. The amount used varied little, from 150 to 160 mg per day, lasting 4 to 8 weeks [26,39,43,51]. Two studies selected epicatechin. Lasting 2 to 4 weeks, doses ranged from 25 to 100 mg per day [39,40,44]. Two studies used epigallocatechin gallate (EGCG), both 8 weeks long and with doses ranging from 300 to 800 mg per day [45,46]. Two other studies utilized genistein, one alone and one as the major isoflavonoid. Both studies were carried out for 8 weeks, and the dose used for both was 50 mg per day [30,47]. A study was conducted with theaflavin and catechin, 75 and 149.4 mg, respectively, for 11 weeks [31]. The last study was conducted mainly with eriocitrin (70%), an eriodictyol glycoside, with doses ranging from 200 to 800 mg per day and a duration of 12 weeks [41] (Table 2).

### 3.5. Outcomes

#### 3.5.1. Anthocyanin-Associated Outcomes

Anthocyanin was used by 11 articles [24,25,27,28,29,32,34,36,37,52,53]. The first was conducted by Qin et al. (2009) with 120 dyslipidemic participants aged 40–65 years who received 120 mg/day of anthocyanin or placebo to assess modifications in lipid profile. In the intervention group there was an increase in HDL-c (+13.7%, *p* < 0.001 vs. placebo) and a reduction in LDL-c (−13.6%, *p* < 0.001), but there were no differences in BW, BMI, WC, SBP, DBP, TC, TG, and glucose [29]. Hassellund et al. (2012 and 2013) conducted two crossover studies for 4 weeks with prehypertensive men aged 35 to 51 years who received 640 mg/day of anthocyanins or placebo [36,37]. The first aimed to assess changes in blood pressure and stress reactivity [36]. Although during the stress test there was a trend towards a reduction in resting supine BP (−6 mmHg), the authors concluded that anthocyanin was not able to significantly alter any parameter when compared to placebo [36]. The second evaluated the effects on cardiovascular risk factors and inflammation [37]. Although there were no changes in levels of TC; LDL-c; HDL-c; TG; insulin; inflammatory markers, such as tumor necrosis factor alpha (TNF-α), high-sensitivity C-reactive protein (hs-CRP), and interleukin-6 (IL-6); and markers of oxidative stress, supplementation caused a significant increase in HDL-c (+0.06 mmol/L, *p* = 0.043 vs. placebo). On the other hand, anthocyanin caused a slight increase in BG (+0.14, *p* = 0.024 vs. placebo) and the von Willebrand factor, a marker of endothelial dysfunction (+5%, *p* = 0.007 vs. placebo) [37]. Three other articles were conducted by Zhu et al. (2011, 2013 and 2014) in subjects with hypercholesterolemia aged 40–65 years who received 320 mg/day of anthocyanin or placebo for 12 [25] or 24 weeks [32,53]. Assessing the endothelial function of 146 subjects, Zhu et al. (2011) reported significant increases in the flow-mediated dilation (FMD) (28.4%, *p* = 0.006 vs. placebo) and HDL-c (+5.8 mg/dL, *p* = 0.010 vs. placebo), as well as decreases in the serum soluble vascular adhesion molecule-1 (sVCAM-1) and LDL-c (−13.5 mg/dL, *p* = 0.015 vs. placebo) [25]. The researchers decided to evaluate, in the same participants, the anti-inflammatory effects of anthocyanin. Anthocyanin consumption significantly decreased the levels of serum hs-CRP (−21.6%, *p* = 0.001 vs. placebo), sVCAM-1 (−12.3%, *p* = 0.005 vs. placebo), and plasma IL-1b (−12.8%, *p* = 0.019 vs. placebo). They also found a significant difference in the LDL-c (−10.4%, *p* = 0.030 vs. baseline) and HDL-c (+14.0%, *p* = 0.036 vs. baseline). No significant differences were observed in the levels of TC and TG [32]. The third one, evaluating 122 subjects also observed significant reduction in LDL-c (−9.72%, *p* < 0.05 vs. placebo) and increase in HDL-c (+11.39%, *p* < 0.05 vs. placebo). However, no differences were observed in TC, triglyceride, glucose, or insulin values [53]. Zhang et al. (2016) evaluated 146 subjects with hypercholesterolemia aged 40–65 years who received 320 mg/day of anthocyanin or placebo for 24 weeks [34]. When compared to the placebo group, individuals who received anthocyanin showed a significant increase in HDL-c (+14%, *p* = 0.036) and reduction in LDL-c (−10.4%, *p* = 0.030), hs-CRP (−21.6%, *p* = 0.001), IL-1β (−12.8%, *p* = 0.019), and sP-selectin (−5.9%, *p* = 0.027). There were no changes in the levels of TC, TG, and TNF-α [34]. One year later, researchers evaluated the ability of anthocyanin supplementation to alleviate thrombogenesis in overweight and obese individuals (28–50 years old) for 4 weeks [52]. With the exception of the results associated with thrombogenesis and platelet activation, such as a reduction in ADP-induced monocyte-platelet aggregate formation by 29% (*p* < 0.05 vs. placebo), there were no differences in glucose, HDL-c, LDL-c, triglyceride, and hs-CRP levels [52]. In their turn, Yang et al. (2020) evaluated 90 prediabetic individuals with a mean age of 60 years who received 320 mg/day of anthocyanin or placebo for 12 weeks [24]. No parameters were significantly changed in the subjects (LDL-c, HDL-c, TC, TG, glucose, or insulin). Despite having evaluated pre and post values, the authors only presented the initial values for BW, BMI, WC, SBP, and DBP [24]. Two studies by Xu et al. (2021) and Zhao et al. (2021) performed the intervention with different doses of anthocyanin (40, 80 or 320 mg/day) in subjects with dyslipidemia with a mean age of 57 years for 12 weeks [27,28]. Xu et al. (2021) reported significant modifications when compared to placebo in subjects receiving 320 mg anthocyanin in cholesterol efflux capacity (+35%, *p* = 0.004 with 320 mg), HDL-c (+0.07 mmol/L, *p* = 0.003 with 320 mg), and ApoA-I (+0.07 g/L with 320 mg, *p* = 0.008), while there was a reduction in all these parameters in the placebo group. There were no differences in anthropometric parameters, such as BW, BMI, and WC, in addition to blood pressure, glucose, and IR. The authors concluded that doses of 80 or 320 mg can improve HDL-c levels and the consequent induction of cholesterol efflux capacity in a dose dependent manner [27]. Zhao et al. (2021) evaluated 169 subjects and like Xu et al. (2021) also reported increased HDL-c (+0.01 ± 0.06 mmol/L, *p* = 0.039), ApoA-I (+0.06 ± 0.08 g/L, *p* = 0.037), and cholesterol efflux capacity (0.25 ± 0.56%, *p* = 0.004) in subjects receiving 320 mg anthocyanin when compared to placebo. In addition, the authors reported reduced plasma ceramides, namely Cer 16:0 and Cer 24:0, were associated in a dose dependent manner with a reduction in non-HDL-c (*p* = 0.040), ApoB (*p* = 0.031), and TC (*p* = 0.026) also in the subjects who received 320 mg [28] (Table 3 and Table 4).

#### 3.5.2. Hesperidin-Associated Outcomes

Six studies selected hesperidin as an intervention [35,38,42,48,49,50]. The first study was conducted with 24 subjects with overweight and moderate hyperlipidemia (age 50–65 years), who for 4 weeks (separated by a 3 weeks washout period) consumed 292 mg/day of hesperidin or placebo [42]. There was no change in anthropometric variables or in the plasma concentrations of glucose and insulin, TG, TC, LDL-c, HDL-c, or in inflammatory markers, such as hs-CRP and IL-6. However, the authors reported significantly reduced DBP, but not SBP, after hesperidin supplementation (−5.3 ± 2.0 mmHg, *p* = 0.023 vs. placebo), and acutely increased the endothelium-dependent vasodilation (+48.65 ± 25.54%, *p* < 0.05 vs. placebo) [42]. In the same year, a study published by Rizza et al. (2011) evaluated the effects of 500 mg/day of hesperidin for 2 periods of 3 weeks separated by a 3 days washout period in 28 subjects (21 and 65 years) with metabolic syndrome [48]. The intervention caused an increase in FMD (+2.47, *p* = 0.02 vs. placebo), although there were no changes in SBP and DBP. In addition, there was a significant reduction in TC (−11.4 mg/dL, *p* = 0.03 vs. placebo), ApoB (−4.79 mg/dL, *p* = 0.04 vs. placebo), and hs-CRP (−0.68 mg/L, *p* = 0.01 vs. placebo), increased HDL-c (+1.3 mg/dL, *p* = 0.05 vs. placebo), and a tendency to increase the quantitative insulin sensitivity check index (QUICKI) (+0.003, *p* = 0.06 vs. placebo). However, hesperidin did not alter WC, body mass index (BMI), and other markers, such as TG, BG, insulin, and LDL-c [48]. From the supplementation of 450 mg/day of hesperidin or placebo, Salden et al. (2016) evaluated endothelial dysfunction in 65 overweight individuals (age 53 ± 14) for 6 weeks [38]. There were no changes in FMD, TC, TG, LDL-c, HDL-c, BG, and insulin; however, the authors reported a trend of reduction (*p* between 0.05 and 0.10) of adhesion molecules, such as sVCAM-1 (*p* = 0.052 vs. placebo), intercellular adhesion molecule 1 (sICAM-1) (*p* = 0.056 vs. placebo), and sP-selectin (*p* = 0.086 vs. placebo), as well as SBP (−5 mmHg, *p* = 0.095 vs. placebo) and DBP (−2 mmHg, *p* = 0.095 vs. placebo) after the six weeks of intervention. In subjects with FMD ≥3%, supplementation caused a significant reduction in sVCAM-1 (*p* = 0.03 vs. placebo) and sICAM-1 (*p* = 0.017 vs. placebo) [38]. Ohara et al. (2016) aimed to evaluate the anti-obesity effect of G-hesperidin or placebo on body fat and serum TG [35]. To do this, they studied 75 healthy subjects (20–65 years) with moderately high body mass index (BMI) and serum TG who received 500 mg/day of G-hesperidin for 12 weeks. There were no differences in BW, BMI, WC, TG, and HDL-c. On the other hand, supplementation was able to reduce TC at week 4 (−12.5 ± 17 mg/dL, *p* < 0.05 vs. baseline) and LDL-c at the end of the study (−10.6 ± 14.0, *p* < 0.05 vs. baseline) [35]. Lastly, two studies by Yari et al. (2020 and 2021) studied the effects of hesperidin in individuals with metabolic syndrome. In the first one, 49 patients with MetS (27–70 years) received either 1000 mg/day hesperidin or placebo for 12 weeks [50]. They evaluated anthropometric parameters, dietary intake, physical activity, lipid profile, glucose homeostasis parameter, TNF-α, and hs-CRP. Hesperidin decreased fasting glucose (−13.32 mg/dL, *p* = 0.043), TG (−49.09 mg/dL, *p* = 0.049), SBP (−2.68 mmHg, *p* = 0.048), and TNF-α (−4.44 pg/mL, *p* = 0.009) and increased QUICKI (+0.02, *p* < 0.001) when compared to placebo. Evaluating pre and post values, Hesperidin was able to reduce the serum levels of glucose (*p* < 0.001), insulin (*p* < 0.001), the homeostasis model assessment of insulin resistance (HOMA-IR) (−0.70, *p* < 0.001) TG (*p* = 0.002), TC (*p* < 0.001), LDL-c (*p* = 0.010), TNF-α (*p* < 0.001) and hs-CRP (*p* = 0.008). Overall, the remission of metabolic syndrome in patients treated with hesperidin was almost twice that of the placebo (64% vs. 33%, *p* = 0.046) [50]. In the second one, evaluating 98 subjects with metabolic syndrome (age ~34–58 years) for 12 weeks who received 1 g/day of hesperidin, they reported significant reduction in SBP (−5.68 mmHg, *p* = 0.041), and serum concentrations of triglyceride (−50.06, *p* = 0.033) when compared to placebo. There was a reduction in DBP pre and post in the group receiving hesperidin (*p* < 0.05), which was not observed in the placebo group. The reduction caused by supplementation of the prevalence of metabolic syndrome was 54.5%, not significant when compared to placebo (−36%). There was no change in BW, BMI, HDL-c, LDL-c, BG and insulin, HOMA-IR, and QUICKI [49] (Table 3 and Table 4).

#### 3.5.3. Quercetin-Associated Outcomes

Four studies used quercetin as an intervention [26,39,43,51]. The first study evaluated the effect of a quercetin supplementation on blood pressure, lipid metabolism, the markers of oxidative stress, inflammation, and body composition in 93 overweight–obese volunteers aged 25–65 years with metabolic syndrome traits [43]. The concentrations of fasting plasma quercetin increased from 71 to 269 nmol/L (349%, *p* < 0.001) during supplementation, which was different compared to placebo values. The authors reported a significant reduction in SBP (−2.6 mmHg in the entire study group, *p* < 0.01 vs. baseline; −2.9 mmHg in the hypertensive subjects, *p* < 0.01 vs. baseline; −3.7 mmHg in the younger adults aged 25–50 years, *p* < 0.001 vs. placebo), in addition to oxidized low-density lipoprotein (ox-LDL) (*p* < 0.001). The supplementation did not significantly affect BW, WC, fat mass, or fat-free mass in the entire study group but decreased the fat mass in women (−0.57 kg, *p* < 0.05). The concentrations of TC, TG, BG, LDL-c, TNF-α, hs-CRP, or uric acid were not influenced, but there was a reduction in HDL-c (−0.07 mmol/L, *p* < 0.001 vs. placebo) [43]. The second study evaluated the same individuals, but they were classified according to apolipoprotein (apo) E genotype into apoE2 (discontinued for low numbers of subjects), apoE3, and apoE4. They also received a dose of 150 mg/day of quercetin or placebo for 2 treatment periods lasting 6 weeks separated by a 5 weeks washout period. The authors reported significant increases in quercetin concentration in subjects in the apoE3 (65.6 to 262.7, +378%, *p* < 0.001 vs. placebo) and apoE4 (89.4 to 293.4, +345%, *p* < 0.001 vs. placebo) groups, with no difference between them. Anthropometric parameters were not influenced by quercetin, such as BW, WC, fat mass, and fat-free mass, as well as some biochemical parameters, such as TC, TG, BG, hs-CRP, and uric acid. Conversely, significant reductions in SBP (−3.4 ± 9.8 mmHg, *p* < 0.01 vs. baseline) but not in DBP were observed in the apoE3 group. Significant reductions were also observed in LDL-c (−0.16 mmol/L, *p* < 0.05 vs. baseline in apoE3), ox-LDL (−186,5 ug/L, *p* < 0.001 vs. baseline in apoE3, −169,8 ug/L, *p* < 0.01 vs. placebo in apoE4), TNF-α (−0.22 ng/L, *p* < 0.01 vs. baseline in apoE3, −0.33 ng/L, *p* < 0.01 vs. baseline in apoE4), and HDL-c (−0.13 mmol/L, *p* < 0.01 vs. baseline in apoE4) [51]. The results of these studies indicate that quercetin may reduce the risk for CVD by reducing blood pressure, TNF-α, and ox-LDL. In a similar study, also dividing participants according to apoE genotype, Pfeuffer et al. (2013) evaluated endothelial function, anthropometry, metabolic, and inflammatory parameters of 49 subjects (age 59.4 ± 0.9) with apoE3 or apoE4 after 150 mg/day quercetin or placebo for 2 treatment periods lasting 8 weeks separated by a 3 weeks washout period [26]. Similar to the previous studies, quercetin reduced SBP, but only postprandial SBP (−5.73 mmHg, *p* < 0.05 in apoE3 and in apoE4 vs. placebo). Fasting SBP and DBP were not altered. Unlike previous studies, quercetin decreased WC (−0.63 cm, *p* < 0.01 in apoE3 and in apoE4 vs. placebo), increased plasma HDL-c levels (+0.06 mmol/L, *p* < 0.05 in apoE3 and in apoE4 vs. placebo) and reduced postprandial triglyceride concentrations in the first 4 h, but not 8 h (−11%, *p* < 0.05 in apoE3 and in apoE4 vs. placebo). BG and insulin levels were not altered. However, quercetin caused a slight increase in TNF-α levels (+11 pg/mL, *p* < 0.05 in apoE3 and in apoE4 vs. placebo). Other inflammatory markers, such as hs-CRP and ox-LDL, were not altered [26]. Lastly, the only study that did not observe any effect associated with quercetin supplementation was conducted by Dower et al. (2015). Of the four studies, this was the one that performed the supplementation for the shortest time (2 treatment periods lasting 4 weeks separated by 4 weeks washout period). The authors evaluated CVD risk factors in 37 apparently healthy men and women aged 40–80 years with a SBP between 125 and 160 mm after 160 mg/day quercetin or placebo. Quercetin supplementation was unable to alter SBP, DBP, BG, IR, HDL-c, LDL-c, or TG, despite significant increases in plasma concentrations after 4 weeks (381 ± 205 nmol/L to 707 ± 517 nmol/L, *p* < 0.001), which was not observed in the control group (393 ± 217 nmol/L, no changes) [39] (Table 3 and Table 4). 

#### 3.5.4. Epicatechin-Associated Outcomes

Three studies evaluated the effects of epicatechin [39,40,44]. Dower et al. (2015) evaluated CVD risk factors in 37 apparently healthy men and women aged 40–80 years with SBP between 125 and 160 mm after 100 mg/day epicatechin or placebo (2 treatment periods lasting 4 weeks separated by 4 weeks washout period). Although the plasma concentration was not detected in fasting samples, 2 h after a supplementation the epicatechin concentrations were 1950 ± 2070 nmol/L. The authors reported significant reductions in mean fasting plasma insulin (−1.46 mU/L, *p* = 0.03 vs. placebo), which, consequently, resulted in a significant decrease in HOMA-IR (−0.38, *p* = 0.04 vs. placebo). However, epicatechin had no effect on any other cardiometabolic health markers, including SBP, DBP, BG, HDL-c, LDL-c, TC, or TG [39]. Esser et al. (2018) evaluated 32 (pre)hypertensive subjects aged 30–80 years that received 2 crossover interventions for 4 weeks with epicatechin (100 mg/day) or placebo with a 4 weeks washout period. The subjects showed significant reduction in plasma glucose (−0.06 ± 0.38 mmol/L, *p* = 0.03 vs. placebo), plasma insulin (−0.80 ± 2.21, *p* = 0.02 vs. placebo), HOMA-IR (−0.23 ± 0.66, *p* = 0.03 vs. placebo), and HOMA-B (−5.1 ± 17.6, *p* = 0.03 vs. placebo). On the other hand, epicatechin did not cause modifications in SBP, DBP, and BW. The inflammation was assessed from gene expression. The authors reported that epicatechin downregulated gene sets involved in inflammation (IL8-CXCR1/2 and AMB2_neutrophil), besides inhibiting upstream regulators involved in inflammation, such as nuclear factor kappa-light-chain-enhancer of activated B cells (NF-κB) (inflammatory type molecules), TNFα and IL-1β (cytokine), although the difference in upstream regulators was not significant when compared to placebo [40]. Thus, the results of the studies indicate that epicatechin may contribute to CVD risk reduction by decreasing IR. As observed with quercetin supplementation, the only study that did not observe differences after epicatechin supplementation performed the supplementation for the shortest period (2 weeks) and supplemented at the lowest dose (25 mg/day) [44]. They evaluated 48 overweight-to-obese subjects with signs of metabolic syndrome for 2 treatment periods lasting 2 weeks separated by 2 weeks washout period. Neither of the next parameters were influenced by supplementation, including blood pressure, BG, insulin, HOMA-IR, TG, LDL-c, HDL-c, ox-LDL, BW, WC fat mass, and fat distribution [44] (Table 3 and Table 4).

#### 3.5.5. Epigallocatechin Gallate-Associated Outcomes

Two studies performed the intervention with EGCC [45,46]. Brown et al. (2009), studied overweight or obese male subjects aged 40–65 years who received 800 mg/day of EGCC or placebo for 8 weeks. They assessed oral glucose tolerance and metabolic risk factors, such as BMI, WC, body fat, blood pressure, TC, LDL-c, HDL-c, and TG. Although EGCC was able to reduce DBP (−2.68 mmHg, *p* = 0.014 vs. placebo), no other parameters were influenced by supplementation [46]. Similarly, Chatree et al. (2021) also reported that EGCC reduced SBP (−6.92 mmHg, *p* = 0.036 vs. baseline) and DBP (−2.00 mmHg, *p* = 0.044 vs. baseline). In addition, there was a significant reduction in plasma triglyceride levels (−29.58 mg/dL, *p* < 0.05 vs. baseline). No other anthropometric (BW, WC, and BMI) or biochemical (BG, insulin, HOMA-IR, TC, HDL-c and LDL-c) parameters were changed. They studied obese subjects who were older than 18 years, and who received 150 mg/day of EGCC or placebo for 8 weeks [45] (Table 3 and Table 4). 

#### 3.5.6. Genistein-Associated Outcomes

Two studies evaluated the effects of genistein [30,47]. The first evaluated lipid concentrations in 45 men and women who received 55 mg isoflavonoids, predominantly genistein (mean age 57 years) or placebo (mean age 54.3 years) for 8 weeks [30]. Despite the higher urinary excretion of genistein in the group receiving the supplement when compared to placebo (*p* < 0.0001), there was no change in lipid and lipoprotein concentrations (TC, LDL-c, HDL-c, tryglicerides, and lipoprotein (a)) [30]. Using 50 mg/day of genistein or placebo for 8 weeks in 45 obese subjects (20–60 years) with a HOMA index greater than 2.5, Guevara-Cruz et al. (2020) studied the gut microbiota and metabolic parameters, especially insulin sensitivity [47]. Although genistein supplementation did not alter BMI, WC, body fat, BW, HDL-c, LDL-c, TG, SBP, DBP, and BG, it did reduce basal insulin (−24%, *p* = 0.05 vs. placebo) and consequent HOMA-IR (−28%, *p* = 0.05 vs. placebo), in addition to a trend of decrease in hs-CRP (*p* = 0.06 vs. placebo). After oral glucose tolerance test (OGTT), a reduction in IR (−19.4%, *p* = 0.02 vs. baseline), and a tendency to increase Matsuda index (*p* = 0.07 vs. baseline) were reported [47] (Table 3 and Table 4).

#### 3.5.7. Theaflavins and Catechin-Associated Outcomes

Trautwein et al. (2010) was the only study that evaluated the effects of theaflavins. They studied the effects of ingesting a purified black tea theaflavins powder alone (77.5 mg) or in combination with catechin (75.0 mg TFs + 149.4 mg catechins) on lowering serum total and LDL-cholesterol in 102 subjects (18 and 65 years) with moderate hypercholesterolemia (TC between 4.80 and 7.00 mmol/L, LDL-c between 2.50 and 4.90 mmol/L and fasting TG < 4.50 mmol/L) for 11 weeks. The authors reported that no evaluated parameters (TC, LDL-c, HDL-c, and TG) were influenced by the supplementation [31] (Table 3 and Table 4). 

#### 3.5.8. Eriocitrin, Hesperidin, Naringin, and Didymin-Associated Outcomes

Another study evaluated the effects of citrus flavonoids [41]. The study evaluated the effects of a supplement (Eriomin^®^) that contains, for the most part, (70%) eriocitrin (eriodictyol glycoside) but also has 5% hesperidin, 4% naringin, and 1% didymin. The study was conducted with 103 prediabetes patients (49 ± 10 years), who received placebo, 200, 400 or 800 mg of Eriomin for 12 weeks. The authors evaluated biochemical, metabolic, inflammatory, hepatic, renal, anthropometric markers, blood pressure, and dietary parameters. The treatment with all doses of Eriomin was effective in reducing the following parameters: BG (−6% with 200 mg, *p* < 0.01 vs. placebo; −5% with 400 mg, *p* < 0.01 vs. placebo; −4% with 800 mg, *p* = 0.041 vs. placebo), IR (−8% with 200 mg, *p* = 0.037 vs. placebo; −7% with 400 mg, *p* = 0.043 vs. placebo; −6% with 800 mg, *p* = 0.042 vs. placebo), glucose intolerance (−7% with 200 or 400 mg, *p* < 0.05 vs. placebo; −6% with 800 mg, *p* < 0.05 vs. placebo), glycated hemoglobin (−2%, *p* < 0.05 vs. placebo), C-peptide (−5%, *p* < 0.001 vs. placebo), hs-CRP (−12%, *p* < 0.05 vs. placebo), interleukin-6 (−13%, *p* = 0.034 vs. placebo), TNFα (−12%, *p* = 0.041 vs. placebo), SBP (−7%, *p* < 0.05 vs. placebo), and antioxidant capacity (+6%, *p* = 0.031 vs. placebo). Anthropometric parameters were not altered by supplementation, such as BW, BMI, lean mass, fat percentage, and hip–waist ratio, in addition to DBP, TC, LDL-c, HDL-c, and TG [41] (Table 3 and Table 4).

## 4. Discussion

In this review study, we aimed to evaluate the ability of flavonoids to affect metabolic parameters that make up the metabolic syndrome, as well as associated parameters, such as inflammation and oxidative stress. From the included studies, this review demonstrated that flavonoids, when used alone as supplements, can significantly modify several metabolic parameters, and consequently reduce the risk of diseases associated with MetS. Of all the flavonoids studied, only theaflavin and catechin were unable to alter metabolic parameters, but only one study was conducted with these compounds. Our conclusion was based on a high number of articles (twenty-nine), all randomized controlled trials, of which eighteen were randomized trials (parallel groups) and the other eleven were crossover trials (individually randomized).

Overall, the following parameters were positively altered by flavonoid supplementation: SBP (57.1%), HDL-c (47.6%), IR (42.8%), LDL-c (40%), DBP (29.4%), TC (17.3%), TG (16%), BG (14.2%), and WC (6.2%). Only BW and BMI were not changed from supplementation. Regarding flavonoids, hesperidin was the one that changed parameters the most, influencing 8 of the 11 evaluated (SBP, DBP, LDL-c, HDL-c, TG, TC, BG, and IR), followed by quercetin (WC, SBP, LDL-c, HDL-c, and TG), anthocyanin (LDL-c, HDL-c, and TC), epigallocatechin gallate (SBP, DBP, and TG), epicatechin (BG and IR), eriocitrin (SBP and BG), and genistein (IR). Importantly, HDL-c was negatively influenced in two studies with quercetin, and BG in one study with anthocyanin. Regarding the two main secondary parameters (TNF-α and hs-CRP), of the nine studies that evaluated TNF-α, a significant reduction in this marker was found in four studies (44.4%, with hesperidin, quercetin, and eriocitrin), however, one study reported an increase associated with quercetin supplementation. The hs-CRP was evaluated in twelve studies and five reported a significant reduction (41.6%, with anthocyanin, hesperidin, genistein, and eriocitrin).

As seen previously, the MetS is a condition that is associated with an increased risk of developing CVD due to changes in metabolic parameters, such as the lipid profile [1,2]. Changes in the lipid profile responsible for increased risk have long been described and are mainly characterized by increased triglyceride and LDL-c levels, but later also associated with reduced HDL-c levels [54,55,56]. Given that HDL-c concentrations are inversely associated with the risk of developing CVD independently of triglyceride and LDL-c levels, the development of strategies aimed at increasing this lipoprotein are critical [55]. The main function of HDL-c involves the reverse transport of cholesterol (RCT), that is, from tissues to the liver [57]. In addition to promoting RCT, HDL-c is also reported to promote systemic anti-inflammatory, antioxidant effects, and protect endothelial cell function [58,59,60]. According to the results found in different publications, the flavonoids anthocyanin, hesperidin, and quercetin caused changes in HDL-c and/or LDL-c levels, while anthocyanin, hesperidin, quercetin, and epigallocatechin gallate were able to alter triglyceride and/or TC levels.

It is important to note that these results are of clinical significance. For each 1% of reduction in LDL or increase in HDL, the CVD event rate is reduced by nearly 1% [61]. The increase in HDL-c levels and reduction in LDL-c caused by anthocyanin in the study conducted by Qin et al. (2009), for example, was 13.7% and 13.6% (27.3% reduction in coronary heart disease risk), respectively. The results may be explained, in part, by a reduction in the mass and activity of cholesteryl ester transfer protein (CETP), a plasma protein that removes cholesteryl esters from HDL in exchange for a triglyceride molecule [29]. In fact, a study using a CETP inhibitor (torcetrapib) had already observed increases in HDL-c levels and decreases in LDL-c [62]. However, an increase in blood pressure and aldosterone concentrations associated with torcetrapib have also been reported [63], which was not found by any studies present in this review that observed positive changes in HDL-c and LDL-c associated with anthocyanin, hesperidin, or quercetin. Importantly, the effects may be associated with the duration and dose of the studies, as addressed by Hassellund et al. (2013) and Xu et al. (2021), who studied anthocyanins. The former found a less pronounced HDL-c increase and no changes in LDL-c after 4 weeks [37], unlike the other studies that lasted 12 weeks [25,29,32,52]. The second, although it observed increased ApoA-I levels with 80 mg per day, did not observe differences in HDL-c levels, which were reported with the highest dose (320 mg/day) [27]. 

Other important mechanisms have been described in studies with anthocyanin and hesperidin. Since the amount of HDL-c alone is not a sufficient parameter to reduce risk [64], studies have evaluated parameters associated with its functionality. The first mechanism is the ability of anthocyanin to increase the level of ApoA-I, the most important structural protein of HDL-c [27]. The HDL-C/apoA-I ratio is already known as a biomarker of HDL-c particles to predict cardiovascular risk [65]. A second mechanism is related to the activity of Paraoxonase 1 (PON1), an enzyme associated with high-density lipoprotein (HDL-PON1) [53]. In this study, in addition to increased HDL-c levels and reduced LDL-c, the authors reported increased HDL-PON1 activity and increased cholesterol efflux capacity associated with anthocyanin supplementation. A negative correlation was observed between HDL-PON1 activity and the levels of lipid hydroperoxides associated with HDL-c, confirming the relationship between the activity of this enzyme and the lipid peroxidation of lipoproteins, although the mechanisms on PON1-mediated antioxidant effects remains to be determined. Furthermore, strong positive correlation was found between HDL-PON1 levels and cholesterol efflux capacity (CEC) [53]. The CEC is considered a significant indicator in the assessment of HDL-C functions, since cholesterol efflux from macrophages is the initial and rate-limiting step of RCT [27]. 

Regarding hesperidin, the activation of peroxisome proliferator-activated receptors (PPAR-alpha), modulation of apoB100 and 3-hydroxy-3-methylglutaryl coenzyme A (HMG-CoA) reductase secretion, and increase in the bile–pancreatic flow are considered potential mechanisms of action in view of, for example, PPAR-alpha showing stimulatory effects on HDL-c and triglyceride concentrations [66,67,68,69,70]. Moreover, apoB-containing lipoproteins overproduction by hepatic tissue is a characteristic of dyslipidemias associated with IR [48]. Ohara et al. (2015) also reported that hesperidin can reduce triglyceride production from inhibition of hepatic lipogenesis [71]. They demonstrated in rats that the inhibition of very-low-density lipoprotein (VLDL) synthesis was from the inhibition of fatty acid synthase (FAS) from the metabolites of hesperidin, which, consequently, would prevent the transformation of malonyl-CoA into palmitic acid and subsequently into TG, which would become available for VLDL production [71].

Two studies in this review found a significant reduction in HDL-c after quercetin supplementation [43,51]. In one of the studies, this reduction was not associated with increased LDL:HDL cholesterol or TAG:HDL cholesterol ratios, thus limiting clinical relevance [43], but was associated with decreased serum HDL cholesterol and apoA1 in apoE4 allele but not in apoE3 carriers, evidencing for the first time phenotype-specific adverse effects [51]. Although the mechanisms have not been elucidated, it may be involved with expression and/or activity of enzymes, such as cholesterol ester transfer protein and lecithin cholesterol acyltransferase or hepatic lipase [51]. Importantly, the latter study also found a reduction in LDL-c, and both studies reported a reduction in ox-LDL [43,51]. The conversion of LDL to ox-LDL is well known to be an atherogenic factor [72], and is found to be elevated in individuals with metabolic syndrome [73]. An in vitro study by Wai et al. (2008) reported the ability of metabolites, such as quercetin-3-O-glucuronide, to inhibit neutrophil-mediated LDL oxidation [74]. In contrast, Pfeuffer et al. (2013) observed increased HDL-c and reduced postprandial TG after quercetin supplementation, with the latter possibly being related to quercetin-induced reduction in fatty acid and triacylglycerol synthesis in the liver [26]. The authors reported that the results were not associated with the different types of phenotypes, as observed by Egert et al. (2010), which can be explained by differences in metabolic conditions of the individuals, gender, and age. Whereas Pfeuffer et al. (2013) evaluated only men, Egert et al. (2009) evaluated both sexes [26,51]. 

Furthermore, EGCG supplementation was also able to alter triglyceride levels. In the only study that observed such a change, the researchers suggested that this result may have been caused by decreased triglyceride absorption in the gastrointestinal tract and/or VLDL production and secretion at the liver [45]. Studies in rats were responsible for allowing such suggestions. The inhibition of pancreatic lipase was observed in a dose-dependent manner from EGCG supplementation, which consequently reduced triglyceride absorption and postprandial triglyceridemia [75]. Another study, performed in rat hepatoma cells reported decreased apoB-100 VLDL secretion, as well as TG [76]. Finally, the only studies that did not observe any modification in LDL-c, HDL-c, TC, and triglyceride levels were performed with epicatechin, genistein, theaflavins plus catechin, and eriocitrin. The supplementation of EGCG, as well as hesperidin, quercetin, and eriocitrin were also able to significantly alter blood pressure.

Both SBP and DBP are used in the criteria for metabolic syndrome (≥130 mmHg SBP or ≥85 mmHg DBP). Although DBP is an important indicator, current literature cites SBP as the main relevant component of blood pressure [77]. The studies in this review reported reductions ranging from 2 to 6 mmHg in DBP and/or SBP. These changes are considered significant because a reduction of 3 to 4 mmHg has already been described in a meta-analysis as capable of reducing the incidence of coronary artery disease by 20% [78]. The mechanisms by which flavonoids may lower blood pressure may involve multiple mechanisms, such as the production of nitric oxide (NO) by vascular endothelium and inhibitory effect on angiotensin-converting enzyme and nicotinamide adenine dinucleotide phosphate (NADPH) oxidase expression [79,80,81]. However, in the study by Morand et al. (2011), where endothelial function was assessed by microvessels by using laser Doppler flowmetry, it was reported, after hesperidin consumption, there was a significant improvement of postprandial acetylcholine-mediated vasodilation and DBP that were not accompanied by changes in plasma NO, raising the possibility of improvements by other mediators, such as prostaglandins [42,82]. 

Other studies have evaluated the ability of flavonoids to alter the function of the vascular endothelium, responsible for maintaining vascular homeostasis through the secretion of vasodilators and vasoconstrictors. Indeed, Rizza et al. (2011) reported improvement in flow-mediated dilation (FMD) but not blood pressure after hesperidin consumption probably associated with higher NO production [48]. On the other hand, Salden et al. (2016) reported that although there were no significant differences in FMD, there was a trend toward a reduction in adhesion molecules, such as sVCAM-1 and sICAM-1, which play an important role in the development and progression of atherosclerosis, as well as DBP and SBP, associated with hesperidin consumption [38]. Thus, the reduction in stressors on the vascular endothelium and consequent improved functionality may also be responsible for higher NO production and subsequent lowering of blood pressure. The reduction in adhesion molecules was significant in a subgroup of subjects with baseline FMD ≥3% [38]. Pfeuffer et al. (2013), meanwhile, found no differences in adhesion molecule concentrations but reported reduced SBP associated with quercetin consumption. The authors suggested that these differences may be associated, in part, with the initial level of endothelial dysfunction [26,38].

Additionally, in a study performed with EGCG, the increase in NO production was caused by the stimulation of phosphotidylinositol-3-kinase/Akt pathway [83]. In another study, EGCG decreased oxidative stress that caused hypertension probably through the scavenging of superoxide anion generation [84]. In one of the studies present in this review, EGCG supplementation also resulted in reduced levels of kisspeptin, which, besides having effects on reproductive function, can be considered a vasoactive substance and actively participate in the regulation of blood pressure [45]. This is because kisspeptin has been revealed to be a potent vasoconstrictor. Its receptor (GPR54) has been found in smooth muscle and endothelial cells, such as human coronary artery, and within atherosclerotic plaque of human coronary artery [85,86]. Taken together, these results confirm the idea that flavonoids act in different ways to improve endothelial function and blood pressure. Due to the existing association between IR and endothelial dysfunction, many studies evaluated the ability of flavonoids to reduce BG and IR. As a matter of fact, by increasing the endothelial bioavailability of NO and consequently decreasing the formation of reactive oxygen and nitrogen species, flavanols may improve IR [87]. 

As proved in some research works analyzed in this review, the flavonoids hesperidin, epicatechin, genistein, and eriocitrin were able to reduce BG and/or IR. The only study that found negative effects was conducted with anthocyanin. However, the authors reported that insulin and long-term glucose (HbA1c) were not significantly different. Furthermore, the analyses were performed 1–3 h after the consumption of anthocyanin capsules containing carbohydrates. Thus, supplementation is unlikely to cause significant negative long-term changes [37]. Moreover, although there are studies demonstrating the effects of this flavonoid on glycemic metabolism, studies suggests that the absence of effects may be due to normal values of fasting BG and HbA1c at baseline [24,27]. In the study by Yari et al. (2020), hesperidin occasioned significant reduction in both parameters [50]. In addition to the bioavailability of NO, several mechanisms have been proposed related to glycemic control. Studying rats with type 2 diabetes, Jung et al. (2006) observed the reduced expression of glucose regulating enzymes genes, such as glucose-6-phosphatase mRNA, phosphoenolpyruvate carboxykinase (PEPCK), as well as increased GLUT4 expression in adipocytes [68]. In another study with rats, Jung et al. (2004) reported that the hypoglycemic effects of hesperidin and naringin are partly mediated by hepatic glucose-regulating enzymes (glycolysis and gluconeogenesis) [88]. Moreover, it is possible that the glycemic control occasioned by hesperidin is attributed to a greater ability to release insulin and consequently greater stimulation for peripheral glucose uptake [89]. In a study with eriodictyol, authors reported regulation in liver and adipocyte expression of peroxisome proliferator-activated receptor gamma (PPARγ) mRNA, which activates insulin signaling and promotes translocation of the glucose transporter GLUT4, which in turn regulates intracellular glucose uptake and consequently insulin sensitivity [90].

The association between HOMA-IR or QUICKI and CVD risk has also been demonstrated in epidemiological studies [91,92]. After supplementation with hesperidin and epicatechin, Rizza et al. (2011) observed a trend toward improvement in insulin sensitivity assessed by QUICKI, while Dower et al. (2015) reported significant reduction in HOMA-IR, respectively [39,48]. In an in vitro study performed with epicatechin, an induction of the AKT/PI-3-kinase and ERK1/2 pathways associated with cocoa flavan-3-ols was observed [93]. In humans, the consumption of high-polyphenol dark chocolate, a source of epicatechin, caused improvement in pancreatic β-cell function [87]. It has been shown that prediabetic individuals may have intestinal alterations that consequently influence glucose and insulin metabolism, such as impaired Glucagon-like peptide 1 (GLP-1) secretion by intestinal L cells after carbohydrate ingestion [94]. Ribeiro et al. (2019), for example, reported a 15% increase in GLP-1 secretion after consumption of citrus flavonoids (mainly eriocitrin), in addition to increased serum adiponectin levels by 18% [41]. GLP-1 regulate glucose metabolism, stimulating insulin secretion, and inhibiting glucagon secretion, as well as adiponectin, which plays a crucial role in insulin sensitivity and the regulation of glucose metabolism [94,95]. 

Guevara-Cruz et al. (2020), one of two studies that used genistein as an intervention, studied the effects on insulin sensitivity through gut microbiota reshaping [47]. Initially, the authors reported reduced IR that may have resulted from increased fatty acid oxidation and increased skeletal muscle oxidative capacity for fatty acids mediated in part by AMPK activation [47]. They then suggested that the effects of genistein on carbohydrate metabolism may occur via a complex mechanism involving the gut microbiota. They found significant increases in the concentrations of the phylum Verrucromicrobia, subsequently associated with an increase in the Akkermansia genus (A. muciniphila at the specie level), which has already been associated with reduced IR [96]. Moreover, they found a reduction in C reactive protein, a marker of systemic inflammation, which has also already been associated with reduced IR [97]. Several studies present in this review evaluated the levels of inflammatory markers, such as hs-CRP and TNF-α, so we included these two markers as secondary endpoints. A significant reduction in TNF-α was found in four studies, while hs-CRP reduction was found in five studies.

Both the high-sensitivity C-reactive protein (hs-CRP) and tumor necrosis factor alpha (TNF-α) are considered markers and regulators of various inflammatory pathways, which contribute to both physiological and pathological processes [98,99]. High levels of these substances are associated with events, such as stroke, myocardial infarction, type 2 diabetes, micro and macrovascular complications, and death [98,100]. In a study by Lee (2011), eriodictyol reduced NO production, suppressed the phagocytic activity of activated macrophages, and reduced the secretion of pro-inflammatory cytokines by reducing the expression of RNA. These repressive effects were caused by inhibition of pathways, such as the nuclear factor kappa-light-chain-enhancer of activated B cells (NF-κB) and the activation of other pathways, such as p38 mitogen-activated protein kinase (MAPK) and extracellular signal-regulated kinases 1 and 2 (ERK1/2) [101]. In another study, Gamo et al. (2014) reported activation of PPARγ and accelerated adipocyte differentiation with a consequent increase in adiponectin with hesperidin [102]. The activation of PPAR is associated with improvement in insulin sensitivity and reduced inflammation, in addition to positive changes in blood pressure [103]. 

Esser et al. (2018), one of the studies evaluated in this review, reported inhibition of upstream regulators, including TNF, although the difference was not significant when compared to placebo [40]. However, based on other studies, the authors hypothesize that epicatechin may act on other inflammatory transcription factors, such as NF-κB and AP-1, as demonstrated by Lee (2011) [101]. Zhang et al. (2016), in another study analyzed in this review, revealed that anthocyanin supplementation caused a significant reduction in several platelet chemokines, such as CXCL7 (secreted only by platelets), which was positively correlated with changes in inflammatory markers, such as hs-CRP, as well as blood lipid levels [34]. Meanwhile, in one of the studies that did not observe significant changes in TNF-α and hs-CRP, Hassellund et al. (2013) suggested that the lack of anti-inflammatory effects may be due to the absence of inflammation or oxidative stress in their study participants at baseline [37]. Thus, it is likely that significant reductions in any parameter evaluated in this review will be obtained if there are significant changes in the first place. Moreover, in view of the association between inflammation, lipid profile, glucose metabolism, and blood pressure, it is possible to conclude that the results found in the studies present a positive correlation between them, as well as there being a complex network connecting the mechanisms of action.

Apart from the many probed beneficial effects of flavonoids, including those found in this review and others, such as appetite control and reduction in food intake, the modulation of adipocyte differentiation, adipogenesis, lipolysis, β-oxidation and apoptosis, stimulation in energy expenditure (thermogenesis), and the modulation of the microbiota, there are some, but not serious adverse effects reported on its use. As pancreatic lipase inhibitors, flavonoids can produce digestive adverse effects, such as abdominal distention or flatulence [104]. From the studies reviewed in this investigation, Hodgson et al. (1998) and Qin et al. (2009) reported no adverse effects with the use of genistein, daidzein, or anthocyanin [29,30]. Esser et al. (2018) studied epicatechin effects and reported only five dropouts as “loss of follow up”, with no adverse events or side effects mentioned [40]. Kirch et al. (2018) used a nutritive dose of epicatechin and also did not report any side effects [44]. Guevara-Cruz et al. (2020) did not report any side effects on genistein treatment on obese subjects [47]. Morand et al. (2011), Rizza et al. (2011), and Yari et al. (2019 and 2020) declare that no adverse events were reported by the study participants during either the placebo or hesperidin treatment period [42,48,49,50].

On the other hand, Egert et al. (2009) reported one drop-out due to gastrointestinal symptoms not related to quercetin supplementation [43]. Xu et al. (2021) reported a total of 18 adverse events with anthocyanins: 4 on placebo group, 6 on 40 mg/day group, 5 on 80 mg/day group, and 3 on 320 mg/day group, ranging from dark stool (most frequent, 10 subjects, 9 under treatment, 1 placebo), insomnia, abdominal pain, diarrhea, dizziness, and skin rash [27]. Zhao et al. (2021) also used anthocyanins at different doses (40, 80, and 320 mg/day). They had a drop-out rate of 10% in each group but claiming several reasons different to adverse or side effects, such as “poor compliance” (*n* = 2), “lost to follow-up” (*n* = 1), “declined to continue” (*n* = 3), or “migrated out” (*n* = 1) [28]. Hassellund et al. (2012 and 2013) reported several adverse events during anthocyanin treatment: diarrhea (*n* = 1), minor headache of short duration (*n* = 3), darker stools (*n* = 2), and nausea (*n* = 1) [36,37]. Yang et al. (2020) also used anthocyanins and reported a total of 10 adverse events among its subjects, 3 of them were of placebo group presenting abdominal pain (*n* = 1), diarrhea (*n* = 1), and skin rash (*n* = 1), while the treated group presented 7 cases with black stool (*n* = 5), insomnia (*n* = 1), and dizziness (*n* = 1) [24]. 

Brown et al. (2009) performed a study with EGCG and reported one subject leaving the protocol due to initiation of drug therapy, but no side effects were mentioned [46]. Chatree et al. (2021) reported in their investigation with EGCG only two adverse events during the treatment: one with headache and the other with a mouth ulcer; however, they mentioned that long term use (>6 months) may be related to hepatic toxicity injury according to other studies [45,105,106]. Trautwein et al. (2010) reported 14 adverse events in its study: 6 occurring in the theaflavins (TFs) group, 2 in the TFs/catechins group, and 6 in the placebo group. Of the 14 adverse events, 13 were not related to the study, while one in the form of stomach discomfort was considered as possibly related to study treatment [31]. Dower et al. (2015) used epicatechin and quercetin and reported two adverse events (one had a nonfatal acute myocardial infarction, while the other had a fatal stroke) but proved to be independent of the treatment [39]. 

Ribeiro et al. (2019) studied Eriomin as the source of flavonoids (mainly eriocitrin) and mentioned seven adverse events during the study: two in the 800 mg/day group, two in the 400 mg group, one in the 200 mg group, and two in the placebo group (pasty stools or headache), but still considered Eriomin well tolerated due to the absence of severe or chronic adverse events [41]. Salden et al. (2016) used hesperidin and only reported one drop-out due to a skin rash, which disappeared after stopping the supplementation [38]. Summarizing, the flavonoid with more reports of adverse events were anthocyanins with gastrointestinal symptoms, such as dark stool, abdominal pain, diarrhea, dizziness, nausea, and others, such as skin rash, minor headache, or insomnia [24,27,28,36,37]. The other flavonoids: catechins, epicatechins and EGCG, hesperidin, quercetin, genistein, and eriocitrin reported occasional and rare side effects among its subjects, and most of them declare not having any adverse events on the studies. 

Nevertheless, natural sources may contain other potentially bioactive compounds that may influence CVD, which casts doubt on whether the beneficial effects on metabolic parameters in humans are solely caused by the intake of polyphenols. Moreover, foods ingested concomitantly may influence degradation and subsequent absorption. The results were also evaluated in individuals with metabolic alterations. Studies with healthy individuals or individuals who do not present alterations in any of the evaluated components may obtain different results, as demonstrated previously. The only study that tested nutritional dosing was unsuccessful, suggesting that higher doses are needed to obtain results, as well as longer-term interventions. Conversely, all the studies evaluated in this review are long-term randomized controlled trials, and only one study was not double-blinded. 

## 5. Conclusions

The results presented in this systematic review offers evidence in support of a flavonoid supplementation, held for at least 3 weeks, as a strategy to improve several metabolic parameters and consequently reduce the risk of diseases associated with MetS. Except for BW and BMI, all other parameters were significantly influenced by flavonoids. This evidence becomes stronger due to the rare side effects reported with flavonoids, except for anthocyanin. Further studies should test different doses of flavonoids as only three studies have evaluated the dose–response effects.

## Figures and Tables

**Table 1:** Characteristics of the included studies and participants.

Authors	Type	Population	Gender	Age
Xu et al., 2021 [27]	Paralell	40 mg (*n* = 45), 80 mg (*n* = 42), 320 mg (*n* = 43), placebo (*n* = 46)	176 total, 26.1% males and 73.9% females	57.41 ± 7.95
Zhu et al., 2011 [25]	Crossover	320 mg (*n* = 75), placebo (*n* = 75)	146 total, 47.8% males and 52.2% females	40–65
Zhu et al., 2014 [52]	Paralell	anthocyanins (*n* = 61), placebo (*n* = 61)	122 total, 42% males and 58% females	55.1 ± 5.4
Zhao et al., 2021 [28]	Paralell	40 mg (*n* = 45), 80 mg (*n* = 42), 320 mg (*n* = 43), placebo (*n* = 46)	169 total, 26.1% males and 73.9% females	57.45 ± 0.86
Thompson et al., 2017 [53]	Paralell	Antocyanins (*n* = 13), placebo (*n* = 13)	26 total, 34.6% males and 65.4% females	39 ± 11
Hassellund et al., 2013 [37]	Crossover	Anthocyanins (*n* = 14), placebo (*n* = 13)	27 males	41 ± 3
Hassellund et al., 2012 [36]	Crossover	Anthocyanins (*n* = 14), placebo (*n* = 13)	27 males	35–51
Qin, et al., 2009 [29]	Paralell	Anthocynins (*n* = 60), placebo (*n* = 60)	120 total, 35% males and 65% females	55.1 ± 5.4 placebo, 55.3 ± 5.0 anthocyanin
Zhu et al., 2013 [32]	Paralell	Anthocyanins (*n* = 73), placebo (*n* = 73)	146 total, 41.8% males and 58.2% females	40–65
Yang et al., 2020 [24]	Paralell	Anthocyanins prediabetes (*n* = 40), placebo prediabetes (*n* = 36)	138 total, 33.75% males and 66.25% females	61.2 ± 6.9 placebo, 60.8 ± 7.9 anthocyanin
Zhang et al., 2016 [34]	Paralell	Anthocyanins (*n* = 73), placebo (*n* = 73)	146 total, 41.8% males and 58.2% females	40–65
Rizza et al., 2011 [48]	Crossover	Hesperidin (*n* = 12), placebo (*n* = 12)	24 total, 62.5% males and 37.5% females	51.66 ± 1.52
Yari et al., 2020 [50]	Paralell	Hesperidin (*n* = 25), placebo (*n* = 24)	49 total, 51% males and 49% females	45.19 ± 11.11
Morand et al., 2011 [42]	Crossover	Control drink + hesperidin (*n* = 8), control drink + placebo (*n* = 8)	24 males	56 ± 1
Salden et al., 2016 [38]	Paralell	Hesperidin (*n* = 34), placebo (*n* = 34)	68 total, 42.6% males and 57.4% females	53 ± 14
Yari et al., 2021 [49]	Paralell	Placebo (*n* = 22), Hesperidin (*n* = 22)	44 total, 47.8% males and 52.2% females	46.41 ± 11.10 control, 45.82 ± 11.69 hesperidin
Ohara et al., 2016 [35]	Paralell	Placebo (*n* = 15), G-Hesperidin (*n* = 15)	74 total, 50% males and 50% females	49.12 ± 1.24
Egert et al., 2009 [43]	Crossover	Quercetin (*n* = 93), placebo (*n* = 93)	93 total, 45.1% males and 54.9% females	45.1 ± 10.53
Egert et al., 2010 [51]	Crossover	apoE3 Quercetin (*n* = 86), E4 Quercetin (86), placebo (*n* = 86)	93 total, 45.1% males and 54.9% females	45.5 ± 9.45
Pfeuffer et al., 2013 [26]	Crossover	apoE3/3 (*n* = 19), apoE4 (*n* = 30)	49 total, 51% males and 49% females	59.4 ± 0.9
Dower et al., 2015 [39]	Crossover	Epicatechin (*n* = 11), Quercetin (*n* = 14), placebo (*n* = 12)	37 total, 67.6% males and 32.4% females	66.4 ± 7.9
Esser et al., 2018 [40]	Crossover	Epicatechin (*n* = 32), placebo (*n* = 32)	32 total, 62.5% males and 37.5% females	65.8 ± 7.9
Kirch et al., 2018 [44]	Crossover	Epicatechin (*n* = 24), placebo (*n* = 23)	47 total, 53.2% males and 46.8% females	36 ± 12 males, 35 ±16 females
Chatree et al., 2021 [45]	Paralell	EGCG (*n* = 15), placebo (*n* = 15)	30, gender no specified	older than 18 years
Brown et al., 2009 [46]	Paralell	EGCG (*n* = 46), placebo (*n* = 42)	88 males	50.57 ± 56.48 placebo 52.15 ± 6.43 EGCG
Guevara-Cruz et al., 2020 [47]	Paralell	Genistein (*n* = 22), placebo (*n* = 23)	45, gender no specified	43.0 ± 2.28 placebo 42.6 ± 1.9 Genistein
Hodgson et al., 1998 [30]	Paralell	Isoflavonoid group (*n* = 30), placebo group (*n* = 29)	59 total, 78% males and 22% females	57.0 placebo and 54.3 Isoflav.
Trautwein et al., 2010 [31]	Paralell	Theaflavins (*n* = 34), Theaflavins + Catechin (*n* = 31) and placebo (*n* = 34)	99 total, 65.7% males and 34.3% females	48.1 ± 6.1
Ribeiro et al., 2019 [41]	Paralell	200 mg (*n* = 26), 400 mg (*n* = 27), 800 mg (*n* = 25), placebo (*n* = 25)	103 total, 46.7% males and 52.4% females	49 ± 10

**Table 2:** Characteristics of the interventions of the included studies.

Authors	Condition	Flavonoid	Dosis	Duration
Xu et al., 2021 [27]	Dyslipidemia	Anthocyanin	40, 80 or 320 mg/day	12 weeks
Zhu et al., 2011 [25]	Hypercholesterolemia	Anthocyanin	320 mg/day	12 weeks
Zhu et al., 2014 [52]	Hypercholesterolemia	Anthocyanin	320 mg/day	24 weeks
Zhao et al., 2021 [28]	Dyslipidemia	Anthocyanin	40, 80 or 320 mg/day	12 weeks
Thompson et al., 2017 [53]	Pro-trombotic overweight and obese individuals	Anthocyanin	320 mg/day	4 weeks
Hassellund et al., 2013 [37]	Prehypertension	Anthocyanin	640 mg/day	4 weeks
Hassellund et al., 2012 [36]	Prehypertension	Anthocyanin	640 mg/day	4 weeks
Qin, et al., 2009 [29]	Dyslipidemia	Anthocyanin	320 mg/day	12 weeks
Zhu et al., 2013 [32]	Hypercholesterolemia	Anthocyanin	320 mg/day	24 weeks
Yang et al., 2020 [24]	Prediabetes	Anthocyanin	320 mg/day	12 weeks
Zhang et al., 2016 [34]	Hypercholesterolemia	Anthocyanin	320 mg/day	24 weeks
Rizza et al., 2011 [48]	Metabolic Syndrome	Hesperidin	500 mg/day	3 weeks
Yari et al., 2020 [50]	Metabolic Syndrome	Hesperidin	1000 mg/day	12 weeks
Morand et al., 2011 [42]	Obesisty	Hesperidin	146 mg/day	4 weeks
Salden et al., 2016 [38]	Overweight prehypertensive	Hesperidin	450 mg/day	6 weeks
Yari et al., 2021 [49]	Metabolic Syndrome	Hesperidin	1000 mg/day	12 weeks
Ohara et al., 2016 [35]	Overweight and hypertriglyceridemia	Glucosyl hesperidin	500 mg/day	12 weeks
Egert et al., 2009 [43]	Overweight or obesity	Quercetin	150 mg/day	6 weeks
Egert et al., 2010 [51]	Metabolic Syndrome	Quercetin	150 mg/day	6 weeks
Pfeuffer et al., 2013 [26]	APOE genotype	Quercetin	150 mg/day	8 weeks
Dower et al., 2015 [39]	Prehypertension	(-)-Epicatechin or Quercetin	epicatechin (100 mg/day) or quercetin-3-glucoside (160 mg/day)	4 weeks
Esser et al., 2018 [40]	Prehypertension	(-)-Epicatechin	100 mg/day	4 weeks
Kirch et al., 2018 [44]	Overweight or obesity and MetS	(-)-Epicatechin	25 mg/day	2 weeks
Chatree et al., 2021 [45]	Obesity	Epigallocatechin gallate	300 mg/day	8 weeks
Brown et al., 2009 [46]	Overweight or obesity	Epigallocatechin gallate	800 mg/day	8 weeks
Guevara-Cruz et al., 2020 [47]	Obesity and insulin resistance	Genistein	50 mg/day	8 weeks
Hodgson et al., 1998 [30]	Predyslipidemia	Isoflavonoids, mainly genistein	55 mg/day	8 weeks
Trautwein et al., 2010 [31]	Dyslipidemia	Theaflavin and catechin	75 mg theaflavins and 149.4 mg catechins	11 weeks
Ribeiro et al., 2019 [41]	Prediabetes	70% eriocitrin, 5% hesperidin, 4% naringin, and 1% didymin	200, 400 or 800 mg/day	12 weeks

**Table 3:** Effects of flavonoids on components of metabolic syndrome (primary outcomes).

Flavonoid	BW	BMI	WC	SBP	DBP	LDL-c	HDL-c	TG	TC	BG	IR
Anthocyanin											
Qin et al., 2009 [29]	=	=	=	=	=	↓	↑	=	=	=	NA
Hassellund et al., 2012 [36]	NA	NA	NA	=	=	NA	NA	NA	NA	NA	NA
Hassellund et al., 2013 [37]	NA	NA	NA	NA	NA	=	↑	=	=	↑	=
Zhu et al., 2011 [25]	=	=	=	=	=	↓	↑	=	=	=	=
Zhu et al., 2013 [32]	NA	NA	NA	NA	NA	↓	↑	=	=	NA	NA
Zhu et al., 2014 [52]	NA	NA	NA	NA	NA	↓	↑	=	=	=	=
Zhang et al., 2016 [34]	NA	NA	NA	NA	NA	↓	↑	=	=	NA	NA
Thompson et al., 2017 [53]	=	=	=	=	=	=	=	=	NA	=	NA
Yang et al., 2020 [24]	NA	NA	NA	NA	NA	=	=	=	=	=	=
Xu et al., 2021 [27]	=	=	=	=	=	=	↑	=	=	=	=
Zhao et al., 2021 [28]	=	=	=	=	=	=	↑	=	↓	=	=
Hesperidin											
Morand et al., 2011 [42]	=	=	NA	=	↓	=	=	=	=	=	=
Rizza et al., 2011 [48]	=	=	=	=	=	=	↑	=	↓	=	↓
Salden et al., 2016 [38]	=	=	=	↓	↓	=	=	=	=	=	=
Ohara et al., 2016 [35]	=	=	=	NA	NA	↓	=	=	↓	NA	NA
Yari et al., 2020 [50]	=	=	=	↓	=	↓	=	↓	↓	↓	↓
Yari et al., 2021 [49]	=	=	=	↓	↓	NA	=	↓	NA	=	=
Quercetin											
Egert et al., 2009 [43]	=	=	=	↓	=	=	↓	=	=	=	NA
Egert et al., 2010 [51]	=	=	=	↓	=	↓	↓	=	=	=	NA
Pfeuffer et al., 2013 [26]	=	=	↓	↓	=	=	↑	↓	=	=	=
Dower et al., 2015 [39]	=	=	NA	=	=	=	=	=	=	=	=
Epicatechin											
Dower et al., 2015 [39]	=	=	NA	=	=	=	=	=	=	=	↓
Esser et al., 2018 [40]	=	=	NA	=	=	=	=	=	=	↓	↓
Kirch et al., 2018 [44]	=	=	=	=	=	=	=	=	=	=	=
Epigallocatechin G.											
Brown et al., 2009 [46]	=	=	=	=	↓	=	=	=	=	=	=
Chatree et al., 2021 [45]	=	=	=	↓	↓	=	=	↓	=	=	=
Genistein											
Hodgson et al., 1998 [30]	=	NA	NA	NA	NA	=	=	=	=	NA	NA
Guevara-Cruz et al., 2020 [47]	=	=	=	=	=	=	=	=	=	=	↓
Theaflavins and Catechin											
Trautwein et al., 2010 [31]	=	=	NA	NA	NA	=	=	=	=	=	NA
Eriocitrin, Hesperidin, Naringin, and Didymin											
Ribeiro et al., 2019 [41]	=	=	NA	↓	=	=	=	=	=	↓	↓

↓↑, significant augmentation or diminution; NA, not assayed; =, no change; BW, body weight; BMI, body mass index; WC, waist circumference; SBP, systolic blood pressure; DBP, diastolic blood pressure; BG, blood glucose; LDL-c and HDL-c, low and high density lipoprotein cholesterol; TG, triglycerides; TC, total cholesterol; IR, insulin resistance.

**Table 4:** Effects of flavonoids on secondary outcomes.

Flavonoid	TNF-α and hs-CRP	Others Markers
Anthocyanin		
Qin et al., 2009 [29]	NA	↓ mass and activity CETP, and ↑ cellular cholesterol efflux
Hassellund et al., 2012 [36]	NA	No changes in levels of renin, aldosterone, or angiotensin-converting enzyme
Hassellund et al., 2013 [37]	No changes in TNF-α and hs-CRP	↑ von Willebrand factor
Zhu et al., 2011 [25]	NA	↓ FMD, cGMP and adhesion molecules, such as sVCAM-1
Zhu et al., 2013 [32]	No changes in TNF-α, ↓ hs-CRP	↓ sVCAM-1 and IL-1β
Zhu et al., 2014 [52]	NA	↑ cholesterol efflux capacity and HDL-PON1 activity
Zhang et al., 2016 [34]	No changes in TNF-α, ↓ hs-CRP	↓ IL-1β and sP-selectin
Thompson et al., 2017 [53]	No changes in hs-CRP	↓ (ADP)-induced platelet activation, PAC-1 and *p*-selectin
Yang et al., 2020 [24]	NA	No changes in adiponectin levels
Xu et al., 2021 [27]	NA	↑ cholesterol efflux capacity and ApoA-I
Zhao et al., 2021 [28]	NA	↑ cholesterol efflux capacity, ApoA-I and Apo B
Hesperidin		
Morand et al., 2011 [42]	No changes in hs-CRP	↑ endothelium-dependent vasodilation and ↓ interleukin-6
Rizza et al., 2011 [48]	↓ hs-CRP	↑ FMD, ↓ apoB, and ↓ sE-selectin
Salden et al., 2016 [38]	NA	No changes in FMD, ↓ adhesion molecules such as sVCAM-1 and sICAM-1
Ohara et al., 2016 [35]	NA	No changes in abdominal fat area (visceral and subcutaneous)
Yari et al., 2020 [50]	↓ TNF-α and hs-CRP	NA
Yari et al., 2021 [49]	NA	NA
Quercetin		
Egert et al., 2009 [43]	No changes in TNF-α and hs-CRP	↓ ox-LDL, ↓ pulse pressure
Egert et al., 2010 [51]	↓ TNF-α, no changes in hs-CRP	↓ ox-LDL
Pfeuffer et al., 2013 [26]	↑TNF-α, no changes in hs-CRP	No changes in s-E-Selectin, s-VCAM, s-ICAM, ox-LDL, and hs-CRP
Dower et al., 2015 [39]	NA	No changes in FMD or EID
Epicatechin		
Dower et al., 2015 [39]	NA	No changes in FMD or EID
Esser et al., 2018 [40]	↓ TNF upstream regulator	↓ IL8-CXCR1/2 and AMB2_neutrophil genes
Kirch et al., 2018 [44]	NA	No changes in ox-LDL
Epigallocatechin G.		
Brown et al., 2009 [46]	NA	↑ mood (hedonic tone)
Chatree et al., 2021 [45]	NA	↓ serum kisspeptin
Genistein		
Hodgson et al., 1998 [30]	NA	No changes in lipoprotein (a) concentrations
Guevara-Cruz et al., 2020 [47]	↓ hs-CRP	↓ metabolic endotoxemia, ↑ skeletal muscle fatty acid oxidation
Theaflavins and Catechin		
Trautwein et al., 2010 [31]	NA	NA
Eriocitrin, Hesperidin, Naringin, and Didymin		
Ribeiro et al., 2019 [41]	↓ TNF-α and hs-CRP	↓ C-peptide and Interleukin-6, ↑ antioxidant capacity

↓↑, Significant augmentation or diminution; NA, not assayed; hs-CRP, highly sensitive C-reactive protein; TNF, tumour necrosis factor; FMD, flow-mediated dilatation; cGMP, cyclic guanosine 3’,5’-monophosphate; sVCAM and VCAM, vascular cell adhesion molecule; IL, interleukin; ICAM, intercellular adhesion molecule; PAC-1, procaspase-activating compound; Apo, apolipoprotein; EID, endothelium-independent dilation; ox-LDL, oxidized low-density lipoprotein; CETP, cholesteryl ester transfer protein.

## Abbreviations

Apo: apolipoprotein; BG, blood glucose; BMI, body mass index; BW, body weight; CETP, cholesteryl ester transfer protein; cGMP, Cyclic guanosine 3’,5’-monophosphate; CVD, cardiovascular disease; DBP, diastolic blood pressure; hs-CRP, highly sensitive C-reactive protein; EID, endothelium-independent dilation; FMD, flow-mediated dilatation; HOMA-IR, homeostasis model assessment of insulin resistance; ICAM, intercellular adhesion molecule; IL, interleukin; IR, insulin resistance; LDL-c and HDL-c, low and high density lipo-protein cholesterol; MetS, metabolic syndrome; MeSH, medical subject headings; ox-LDL, oxidized low-density lipo-protein; PAC-1, procaspase-activating compound; QUICKI, quantitative insulin sensitivity check index; SBP, systolic blood pressure; sP-selectin, soluble platelet selectin; sVCAM and VCAM, vascular cell adhesion molecule; TC, total cholesterol; TG, triglycerides; TNF, tumour necrosis factor; WC, waist circumference.

## References

[1] Grundy S.M., Cleeman J.I., Daniels S.R., Donato K.A., Eckel R.H., Franklin B.A., Gordon D.J., Krauss R.M., Savage P.J., Smith S.C. (2005). Diagnosis and Management of the Metabolic Syndrome: An American Heart Association/National Heart, Lung, and Blood Institute Scientific Statement. Circulation.

[2] Alberti K.G.M.M., Eckel R.H., Grundy S.M., Zimmet P.Z., Cleeman J.I., Donato K.A., Fruchart J.C., James W.P.T., Loria C.M., Smith S.C. (2009). Harmonizing the Metabolic Syndrome: A Joint Interim Statement of the International Diabetes Federation Task Force on Epidemiology and Prevention; National Heart, Lung, and Blood Institute; American Heart Association; World Heart Federation; International Atherosclerosis Society; and International Association for the Study of Obesity. Circulation.

[3] Ranasinghe P., Mathangasinghe Y., Jayawardena R., Hills A.P., Misra A. (2017). Prevalence and Trends of Metabolic Syndrome among Adults in the Asia-Pacific Region: A Systematic Review. BMC Public Health.

[4] Amiot M.J., Riva C., Vinet A. (2016). Effects of Dietary Polyphenols on Metabolic Syndrome Features in Humans: A Systematic Review. Obes. Rev..

[5] do Vale Moreira N.C., Hussain A., Bhowmik B., Mdala I., Siddiquee T., Fernandes V.O., Montenegro Júnior R.M., Meyer H.E. (2020). Prevalence of Metabolic Syndrome by Different Definitions, and Its Association with Type 2 Diabetes, Pre-Diabetes, and Cardiovascular Disease Risk in Brazil. Diabetes Metab. Syndr. Clin. Res. Rev..

[6] Després J.P., Lemieux I. (2006). Abdominal Obesity and Metabolic Syndrome. Nature.

[7] World Health Organization WHO Reveals Leading Causes of Death and Disability Worldwide: 2000–2019. https://www.who.int/news/item/09-12-2020-who-reveals-leading-causes-of-death-and-disability-worldwide-2000-2019.

[8] Paley C.A., Johnson M.I. (2018). Abdominal Obesity and Metabolic Syndrome: Exercise as Medicine?. BMC Sports Sci. Med. Rehabil..

[9] Martínez-González M.A., Salas-Salvadó J., Estruch R., Corella D., Fitó M., Ros E. (2015). Benefits of the Mediterranean Diet: Insights From the PREDIMED Study. Prog. Cardiovasc. Dis..

[10] Finicelli M., Squillaro T., Di Cristo F., Di Salle A., Melone M.A.B., Galderisi U., Peluso G. (2019). Metabolic Syndrome, Mediterranean Diet, and Polyphenols: Evidence and Perspectives. J. Cell. Physiol..

[11] Sohrab G., Ebrahimof S., Hosseinpour-Niazi S., Yuzbashian E., Mirmiran P., Azizi F. (2018). Association of Dietary Intakes of Total Polyphenol and Its Subclasses with the Risk of Metabolic Syndrome: Tehran Lipid and Glucose Study. Metab. Syndr. Relat. Disord..

[12] Lecour S., Lamont K.T. (2012). Natural Polyphenols and Cardioprotection. Mini-Rev. Med. Chem..

[13] Singla R.K., Dubey A.K., Garg A., Sharma R.K., Fiorino M., Ameen S.M., Haddad M.A., Al-Hiary M. (2019). Natural Polyphenols: Chemical Classification, Definition of Classes, Subcategories, and Structures. J. AOAC Int..

[14] Panche A.N., Diwan A.D., Chandra S.R. (2016). Flavonoids: An Overview. J. Nutr. Sci..

[15] Macready A.L., Kennedy O.B., Ellis J.A., Williams C.M., Spencer J.P.E., Butler L.T. (2009). Flavonoids and Cognitive Function: A Review of Human Randomized Controlled Trial Studies and Recommendations for Future Studies. Genes Nutr..

[16] Wang L., Lee I.M., Zhang S.M., Blumberg J.B., Buring J.E., Sesso H.D. (2009). Dietary Intake of Selected Flavonols, Flavones, and Flavonoid-Rich Foods and Risk of Cancer in Middle-Aged and Older Women. Am. J. Clin. Nutr..

[17] Cassidy A., O’Reilly É.J., Kay C., Sampson L., Franz M., Forman J.P., Curhan G., Rimm E.B. (2011). Habitual Intake of Flavonoid Subclasses and Incident Hypertension in Adults. Am. J. Clin. Nutr..

[18] Hertog M., Kromhout D., Aravanis C., Blackburn H., Buzina R., Fidanza F., Giampaoli S., Jansen A., Menotti A., Nedeljkovic S. (1995). Flavonoid Intake and Long-Term Risk in the Seven Countries Study. Arch. Intern Med..

[19] Mink P.J., Scrafford C.G., Barraj L.M., Harnack L., Hong C.P., Nettleton J.A., Jacobs D.R. (2007). Flavonoid Intake and Cardiovascular Disease Mortality: A Prospective Study in Postmenopausal Women. Am. J. Clin. Nutr..

[20] Akbari M., Tamtaji O.R., Lankarani K.B., Tabrizi R., Dadgostar E., Haghighat N., Kolahdooz F., Ghaderi A., Mansournia M.A., Asemi Z. (2020). The Effects of Resveratrol on Lipid Profiles and Liver Enzymes in Patients with Metabolic Syndrome and Related Disorders: A Systematic Review and Meta-Analysis of Randomized Controlled Trials. Lipids Health Dis..

[21] Moher D., Liberati A., Tetzlaff J., Altman D.G. (2009). Reprint-preferred reporting items for systematic reviews and meta-analyses: The PRISMA statement. Phys. Ther..

[22] Sterne J.A.C., Savović J., Page M.J., Elbers R.G., Blencowe N.S., Boutron I., Cates C.J., Cheng H.Y., Corbett M.S., Eldridge S.M. (2019). RoB 2: A Revised Tool for Assessing Risk of Bias in Randomised Trials. BMJ.

[23] Page M.J., McKenzie J.E., Bossuyt P.M., Boutron I., Hoffmann T.C., Mulrow C.D., Shamseer L., Tetzlaff J.M., Akl E.A., Brennan S.E. (2021). The PRISMA 2020 Statement: An Updated Guideline for Reporting Systematic Reviews. BMJ.

[24] Yang L., Ling W., Qiu Y., Liu Y., Wang L., Yang J., Wang C., Ma J. (2020). Anthocyanins Increase Serum Adiponectin in Newly Diagnosed Diabetes but Not in Prediabetes: A Randomized Controlled Trial. Nutr. Metab..

[25] Zhu Y., Xia M., Yang Y., Liu F., Li Z., Hao Y., Mi M., Jin T., Ling W. (2011). Purified Anthocyanin Supplementation Improves Endothelial Function via NO-CGMP Activation in Hypercholesterolemic Individuals. Clin. Chem..

[26] Pfeuffer M., Auinger A., Bley U., Kraus-Stojanowic I., Laue C., Winkler P., Rüfer C.E., Frank J., Bösch-Saadatmandi C., Rimbach G. (2013). Effect of Quercetin on Traits of the Metabolic Syndrome, Endothelial Function and Inflammation in Men with Different APOE Isoforms. Nutr. Metab. Cardiovasc. Dis..

[27] Xu Z., Xie J., Zhang H., Pang J., Li Q., Wang X., Xu H., Sun X., Zhao H., Yang Y. (2021). Anthocyanin Supplementation at Different Doses Improves Cholesterol Efflux Capacity in Subjects with Dyslipidemia—A Randomized Controlled Trial. Eur. J. Clin. Nutr..

[28] Zhao Y., Xu H., Tian Z., Wang X., Xu L., Li K., Gao X., Fan D., Ma X., Ling W. (2021). Dose-Dependent Reductions in Plasma Ceramides after Anthocyanin Supplementation Are Associated with Improvements in Plasma Lipids and Cholesterol Efflux Capacity in Dyslipidemia: A Randomized Controlled Trial. Clin. Nutr..

[29] Qin Y., Xia M., Ma J., Hao Y., Liu J., Mou H., Cao L., Ling W. (2009). Anthocyanin Supplementation Improves Serum LDL- and HDL-Cholesterol Concentrations Associated with the Inhibition of Cholesteryl Ester Transfer Protein in Dyslipidemic Subjects. Am. J. Clin. Nutr..

[30] Hodgson J.M., Puddey I.B., Beilin L.J., Mori T.A., Croft K.D. (1998). Supplementation with Isoflavonoid Phytoestrogens Does Not Alter Serum Lipid Concentrations: A Randomized Controlled Trial in Humans. J. Nutr..

[31] Trautwein E.A., Du Y., Meynen E., Yan X., Wen Y., Wang H., Molhuizen H.O.F. (2010). Purified Black Tea Theaflavins and Theaflavins/Catechin Supplements Did Not Affect Serum Lipids in Healthy Individuals with Mildly to Moderately Elevated Cholesterol Concentrations. Eur. J. Nutr..

[32] Zhu Y., Ling W., Guo H., Song F., Ye Q., Zou T., Li D., Zhang Y., Li G., Xiao Y. (2013). Anti-Inflammatory Effect of Purified Dietary Anthocyanin in Adults with Hypercholesterolemia: A Randomized Controlled Trial. Nutr. Metab. Cardiovasc. Dis..

[33] Qin Y., Shu F., Zeng Y., Meng X., Wang B., Diao L., Wang L., Wan J., Zhu J., Wang J. (2014). Daidzein Supplementation Decreases Serum Triglyceride and Uric Acid Concentrations in Hypercholesterolemic Adults with the Effect on Triglycerides Being Greater in Those with the GA Compared with the GG Genotype of ESR-β RsaI. J. Nutr..

[34] Zhang X., Zhu Y., Song F., Yao Y., Ya F., Li D., Ling W., Yang Y. (2016). Effects of Purified Anthocyanin Supplementation on Platelet Chemokines in Hypocholesterolemic Individuals: A Randomized Controlled Trial. Nutr. Metab..

[35] Ohara T., Muroyama K., Yamamoto Y., Murosaki S. (2016). Oral Intake of a Combination of Glucosyl Hesperidin and Caffeine Elicits an Anti-Obesity Effect in Healthy, Moderately Obese Subjects: A Randomized Double-Blind Placebo-Controlled Trial. Nutr. J..

[36] Hassellund S.S., Flaa A., Sandvik L., Kjeldsen S.E., Rostrup M. (2012). Effects of Anthocyanins on Blood Pressure and Stress Reactivity: A Double-Blind Randomized Placebo-Controlled Crossover Study. J. Hum. Hypertens..

[37] Hassellund S.S., Flaa A., Kjeldsen S.E., Seljeflot I., Karlsen A., Erlund I., Rostrup M. (2013). Effects of Anthocyanins on Cardiovascular Risk Factors and Inflammation in Pre-Hypertensive Men: A Double-Blind Randomized Placebo-Controlled Crossover Study. J. Hum. Hypertens..

[38] Salden B.N., Troost F.J., De Groot E., Stevens Y.R., Garcés-Rimón M., Possemiers S., Winkens B., Masclee A.A. (2016). Randomized Clinical Trial on the Efficacy of Hesperidin 2S on Validated Cardiovascular Biomarkers in Healthy Overweight Individuals. Am. J. Clin. Nutr..

[39] Dower J.I., Geleijnse J.M., Gijsbers L., Zock P.L., Kromhout D., Hollman P.C.H. (2015). Effects of the Pure Flavonoids Epicatechin and Quercetin on Vascular Function and Cardiometabolic Health: A Randomized, Double-Blind, Placebo-Controlled, Crossover Trial. Am. J. Clin. Nutr..

[40] Esser D., Geleijnse J.M., Matualatupauw J.C., Dower J.I., Kromhout D., Hollman P.C.H., Afman L.A. (2018). Pure Flavonoid Epicatechin and Whole Genome Gene Expression Profiles in Circulating Immune Cells in Adults with Elevated Blood Pressure: A Randomised Double-Blind, Placebo-Controlled, Crossover Trial. PLoS ONE.

[41] Ribeiro C.B., Ramos F.M., Manthey J.A., Cesar T.B. (2019). Effectiveness of Eriomin® in Managing Hyperglycemia and Reversal of Prediabetes Condition: A Double-Blind, Randomized, Controlled Study. Phyther. Res..

[42] Morand C., Dubray C., Milenkovic D., Lioger D., Martin J.F., Scalbert A., Mazur A. (2011). Hesperidin Contributes to the Vascular Protective Effects of Orange Juice: A Randomized Crossover Study in Healthy Volunteers. Am. J. Clin. Nutr..

[43] Egert S., Bosy-Westphal A., Seiberl J., Kuerbitz C., Settler U., Plachta-Danielzik S., Wagner A.E., Frank J., Schrezenmeir J., Rimbach G. (2009). Quercetin Reduces Systolic Blood Pressure and Plasma Oxidised Low-Density Lipoprotein Concentrations in Overweight Subjects with a High-Cardiovascular Disease Risk Phenotype: A Double-Blinded, Placebo-Controlled Cross-over Study. Br. J. Nutr..

[44] Kirch N., Berk L., Liegl Y., Adelsbach M., Zimmermann B.F., Stehle P., Stoffel-Wagner B., Ludwig N., Schieber A., Helfrich H.-P. (2018). A Nutritive Dose of Pure (-)-Epicatechin Does Not Beneficially Affect Increased Cardiometabolic Risk Factors in Overweight-to-Obese Adults—A Randomized, Placebo-Controlled, Double-Blind Crossover Study. Am. J. Clin. Nutr..

[45] Chatree S., Sitticharoon C., Maikaew P., Pongwattanapakin K., Keadkraichaiwat I., Churintaraphan M., Sripong C., Sririwichitchai R., Tapechum S. (2021). Epigallocatechin Gallate Decreases Plasma Triglyceride, Blood Pressure, and Serum Kisspeptin in Obese Human Subjects. Exp. Biol. Med..

[46] Brown A.L., Lane J., Coverly J., Stocks J., Jackson S., Stephen A., Bluck L., Coward A., Hendrickx H. (2009). Effects of Dietary Supplementation with the Green Tea Polyphenol Epigallocatechin-3-Gallate on Insulin Resistance and Associated Metabolic Risk Factors: Randomized Controlled Trial. Br. J. Nutr..

[47] Guevara-Cruz M., Godinez-Salas E.T., Sanchez-Tapia M., Torres-Villalobos G., Pichardo-Ontiveros E., Guizar-Heredia R., Arteaga-Sanchez L., Gamba G., Mojica-Espinosa R., Schcolnik-Cabrera A. (2020). Genistein Stimulates Insulin Sensitivity through Gut Microbiota Reshaping and Skeletal Muscle AMPK Activation in Obese Subjects. BMJ Open Diabetes Res. Care.

[48] Rizza S., Muniyappa R., Iantorno M., Kim J., Chen H., Pullikotil P., Senese N., Tesauro M., Lauro D., Cardillo C. (2011). Citrus Polyphenol Hesperidin Stimulates Production of Nitric Oxide in Endothelial Cells While Improving Endothelial Function and Reducing Inflammatory Markers in Patients with Metabolic Syndrome. J. Clin. Endocrinol. Metab..

[49] Yari Z., Cheraghpour M., Hekmatdoost A. (2021). Flaxseed and/or Hesperidin Supplementation in Metabolic Syndrome: An Open-Labeled Randomized Controlled Trial. Eur. J. Nutr..

[50] Yari Z., Movahedian M., Imani H., Alavian S.M., Hedayati M., Hekmatdoost A. (2020). The Effect of Hesperidin Supplementation on Metabolic Profiles in Patients with Metabolic Syndrome: A Randomized, Double-Blind, Placebo-Controlled Clinical Trial. Eur. J. Nutr..

[51] Egert S., Boesch-Saadatmandi C., Wolffram S., Rimbach G., Müller M.J. (2010). Serum Lipid and Blood Pressure Responses to Quercetin Vary in Overweight Patients by Apolipoprotein E Genotype. J. Nutr..

[52] Zhu Y., Huang X., Zhang Y., Wang Y., Liu Y., Sun R., Xia M. (2014). Anthocyanin Supplementation Improves HDL-Associated Paraoxonase 1 Activity and Enhances Cholesterol Efflux Capacity in Subjects With Hypercholesterolemia. J. Clin. Endocrinol. Metab..

[53] Thompson K., Pederick W., Singh I., Santhakumar A.B. (2017). Anthocyanin Supplementation in Alleviating Thrombogenesis in Overweight and Obese Population: A Randomized, Double-Blind, Placebo-Controlled Study. J. Funct. Foods.

[54] Assmann G., Schulte H. (1992). Relation of High-Density Lipoprotein Cholesterol and Triglycerides to Incidence of Atherosclerotic Coronary Artery Disease (the PROCAM Experience). Am. J. Cardiol..

[55] Lewis G.F., Rader D.J. (2005). New Insights into the Regulation of HDL Metabolism and Reverse Cholesterol Transport. Circ. Res..

[56] Nicholls S.J., Nelson A.J. (2019). HDL and Cardiovascular Disease. Pathology.

[57] Fielding C.J., Fielding P.E. (1995). Molecular Physiology of Reverse Cholesterol Transport. J. Lipid Res..

[58] Barter P.J., Nicholls S., Rye K.A., Anantharamaiah G.M., Navab M., Fogelman A.M. (2004). Antiinflammatory Properties of HDL. Circ. Res..

[59] deGoma E.M., DeGoma R.L., Rader D.J. (2008). Beyond High-Density Lipoprotein Cholesterol Levels. J. Am. Coll. Cardiol..

[60] Durrington P.N., Mackness B., Mackness M.I. (2001). Paraoxonase and Atherosclerosis. Arterioscler. Thromb. Vasc. Biol..

[61] Brown B.G., Stukovsky K.H., Zhao X.Q. (2006). Simultaneous Low-Density Lipoprotein-C Lowering and High-Density Lipoprotein-C Elevation for Optimum Cardiovascular Disease Prevention with Various Drug Classes, and Their Combinations: A Meta-Analysis of 23 Randomized Lipid Trials. Curr. Opin. Lipidol..

[62] Brousseau M.E., Schaefer E.J., Wolfe M.L., Bloedon L.T., Digenio A.G., Clark R.W., Mancuso J.P., Rader D.J. (2004). Effects of an Inhibitor of Cholesteryl Ester Transfer Protein on HDL Cholesterol. N. Engl. J. Med..

[63] Rader D.J. (2007). Illuminating HDL—Is It Still a Viable Therapeutic Target?. N. Engl. J. Med..

[64] Khera A.V., Cuchel M., de la Llera-Moya M., Rodrigues A., Burke M.F., Jafri K., French B.C., Phillips J.A., Mucksavage M.L., Wilensky R.L. (2011). Cholesterol Efflux Capacity, High-Density Lipoprotein Function, and Atherosclerosis. N. Engl. J. Med..

[65] Sung K.C., Wild S.H., Byrne C.D. (2013). Controlling for Apolipoprotein A-I Concentrations Changes the Inverse Direction of the Relationship between High HDL-C Concentration and a Measure of Pre-Clinical Atherosclerosis. Atherosclerosis.

[66] Assini J.M., Mulvihill E.E., Huff M.W. (2013). Citrus Flavonoids and Lipid Metabolism. Curr. Opin. Lipidol..

[67] Duval C., Müller M., Kersten S. (2007). PPARα and Dyslipidemia. Biochim. Biophys. Acta Mol. Cell Biol. Lipids.

[68] Jung U.J., Lee M.K., Park Y.B., Kang M.A., Choi M.S. (2006). Effect of Citrus Flavonoids on Lipid Metabolism and Glucose-Regulating Enzyme MRNA Levels in Type-2 Diabetic Mice. Int. J. Biochem. Cell Biol..

[69] Akiyama S., Katsumata S.I., Suzuki K., Nakaya Y., Ishimi Y., Uehara M. (2009). Hypoglycemic and Hypolipidemic Effects of Hesperidin and Cyclodextrin-Clathrated Hesperetin in Goto-Kakizaki Rats with Type 2 Diabetes. Biosci. Biotechnol. Biochem..

[70] Gorinstein S., Caspi A., Libman I., Leontowicz H., Leontowicz M., Tashma Z., Katrich E., Jastrzebski Z., Trakhtenberg S. (2007). Bioactivity of Beer and Its Influence on Human Metabolism. Int. J. Food Sci. Nutr..

[71] Ohara T., Muroyama K., Yamamoto Y., Murosaki S. (2015). A Combination of Glucosyl Hesperidin and Caffeine Exhibits an Anti-Obesity Effect by Inhibition of Hepatic Lipogenesis in Mice. Phyther. Res..

[72] Stocker R., Keaney J.F. (2004). Role of Oxidative Modifications in Atherosclerosis. Physiol. Rev..

[73] Holvoet P., Kritchevsky S.B., Tracy R.P., Mertens A., Rubin S.M., Butler J., Goodpaster B., Harris T.B. (2004). The Metabolic Syndrome, Circulating Oxidized LDL, and Risk of Myocardial Infarction in Well-Functioning Elderly People in the Health, Aging, and Body Composition Cohort. Diabetes.

[74] Wai M.L., Proudfoot J.M., Mckinley A.J., Needs P.W., Kroon P.A., Hodgson J.M., Croft K.D. (2008). Quercetin and Its in Vivo Metabolites Inhibit Neutrophil-Mediated Low-Density Lipoprotein Oxidation. J. Agric. Food Chem..

[75] Ikeda I., Tsuda K., Suzuki Y., Kobayashi M., Unno T., Tomoyori H., Goto H., Kawata Y., Imaizumi K., Nozawa A. (2005). Tea Catechins with a Galloyl Moiety Suppress Postprandial Hypertriacylglycerolemia by Delaying Lymphatic Transport of Dietary Fat in Rats. J. Nutr..

[76] Li L., Stillemark-Billton P., Beck C., Boström P., Andersson L., Rutberg M., Ericsson J., Magnusson B., Marchesan D., Ljungberg A. (2006). Epigallocatechin Gallate Increases the Formation of Cytosolic Lipid Droplets and Decreases the Secretion of ApoB-100 VLDL. J. Lipid Res..

[77] Franklin S.S. (2004). Systolic Blood Pressure: It’s Time to Take Control. Am. J. Hypertens..

[78] Law M.R., Morris J.K., Wald N.J. (2009). Use of Blood Pressure Lowering Drugs in the Prevention of Cardiovascular Disease: Meta-Analysis of 147 Randomised Trials in the Context of Expectations from Prospective Epidemiological Studies. BMJ.

[79] Grassi D., Desideri G., Croce G., Tiberti S., Aggio A., Ferri C. (2009). Flavonoids, Vascular Function and Cardiovascular Protection. Curr. Pharm. Des..

[80] Fraga C.G., Actis-Goretta L., Ottaviani J.I., Carrasquedo F., Lotito S.B., Lazarus S., Schmitz H.H., Keen C.L. (2005). Regular Consumption of a Flavanol-Rich Chocolate Can Improve Oxidant Stress in Young Soccer Players. Clin. Dev. Immunol..

[81] Yamamoto M., Suzuki A., Jokura H., Yamamoto N., Hase T. (2008). Glucosyl Hesperidin Prevents Endothelial Dysfunction and Oxidative Stress in Spontaneously Hypertensive Rats. Nutrition.

[82] Turner J., Belch J.J.F., Khan F. (2008). Current Concepts in Assessment of Microvascular Endothelial Function Using Laser Doppler Imaging and Iontophoresis. Trends Cardiovasc. Med..

[83] Lorenz M., Wessler S., Follmann E., Michaelis W., Düsterhöft T., Baumann G., Stangl K., Stangl V. (2004). A Constituent of Green Tea, Epigallocatechin-3-Gallate, Activates Endothelial Nitric Oxide Synthase by a Phosphatidylinositol-3-OH-Kinase-, CAMP-Dependent Protein Kinase-, and Akt-Dependent Pathway and Leads to Endothelial-Dependent Vasorelaxation. J. Biol. Chem..

[84] Antonello M., Montemurro D., Bolognesi M., Dipascoli M., Piva A., Grego F., Sticchi D., Giuliani L., Garbisa S., Rossi G. (2007). Prevention of Hypertension, Cardiovascular Damage and Endothelial Dysfunction with Green Tea Extracts. Am. J. Hypertens..

[85] Mead E.J., Maguire J.J., Kuc R.E., Davenport A.P. (2007). Kisspeptins Are Novel Potent Vasoconstrictors in Humans, with a Discrete Localization of Their Receptor, G Protein-Coupled Receptor 54, to Atherosclerosis-Prone Vessels. Endocrinology.

[86] Sawyer I., Smillie S.J., Bodkin J.V., Fernandes E., O’Byrne K.T., Brain S.D. (2011). The Vasoactive Potential of Kisspeptin-10 in the Peripheral Vasculature. PLoS ONE.

[87] Grassi D., Desideri G., Necozione S., Lippi C., Casale R., Properzi G., Blumberg J.B., Ferri C. (2008). Blood Pressure Is Reduced and Insulin Sensitivity Increased in Glucose-Intolerant, Hypertensive Subjects after 15 Days of Consuming High-Polyphenol Dark Chocolate. J. Nutr..

[88] Jung U.J., Lee M.K., Jeong K.S., Choi M.S. (2004). The Hypoglycemic Effects of Hesperidin and Naringin Are Partly Mediated by Hepatic Glucose-Regulating Enzymes in C57BL/KsJ-Db/Db Mice. J. Nutr..

[89] Ahmed O.M., Mahmoud A.M., Abdel-Moneim A., Ashour M.B. (2012). Antidiabetic Effects of Hesperidin and Naringin in Type 2 Diabetic Rats. Diabetol. Croat..

[90] Zhang W.Y., Lee J.J., Kim Y., Kim I.S., Han J.H., Lee S.G., Ahn M.J., Jung S.H., Myung C.S. (2012). Effect of Eriodictyol on Glucose Uptake and Insulin Resistance in Vitro. J. Agric. Food Chem..

[91] Bonora E., Kiechl S., Willeit J., Oberhollenzer F., Egger G., Meigs J.B., Bonadonna R.C., Muggeo M. (2007). Insulin Resistance as Estimated by Homeostasis Model Assessment Predicts Incident Symptomatic Cardiovascular Disease in Caucasian Subjects from the General Population: The Bruneck Study. Diabetes Care.

[92] Katz A., Nambi S.S., Mather K., Baron A.D., Follmann D.A., Sullivan G., Quon M.J. (2000). Quantitative Insulin Sensitivity Check Index: A Simple, Accurate Method for Assessing Insulin Sensitivity in Humans. J. Clin. Endocrinol. Metab..

[93] Granado-Serrano A.B., Martín M.A., Haegeman G., Goya L., Bravo L., Ramos S. (2010). Epicatechin Induces NF-ΚB, Activator Protein-1 (AP-1) and Nuclear Transcription Factor Erythroid 2p45-Related Factor-2 (Nrf2) via Phosphatidylinositol-3-Kinase/Protein Kinase B (PI3K/AKT) and Extracellular Regulated Kinase (ERK) Signalling in HepG2 Cells. Br. J. Nutr..

[94] Wang X.L., Ye F., Li J., Zhu L.Y., Feng G., Chang X.Y., Sun K. (2016). Impaired Secretion of Glucagon-like Peptide 1 during Oral Glucose Tolerance Test in Patients with Newly Diagnosed Type 2 Diabetes Mellitus. Saudi Med. J..

[95] Gastaldelli A., Gaggini M., DeFronzo R. (2017). Glucose Kinetics: An Update and Novel Insights into Its Regulation by Glucagon and GLP-1. Curr. Opin. Clin. Nutr. Metab. Care.

[96] Depommier C., Everard A., Druart C., Plovier H., Van Hul M., Vieira-Silva S., Falony G., Raes J., Maiter D., Delzenne N.M. (2019). Supplementation with Akkermansia Muciniphila in Overweight and Obese Human Volunteers: A Proof-of-Concept Exploratory Study. Nat. Med..

[97] Lee W.Y., Park J.S., Noh S.Y., Rhee E.J., Sung K.C., Kim B.S., Kang J.H., Kim S.W., Lee M.H., Park J.R. (2004). C-Reactive Protein Concentrations Are Related to Insulin Resistance and Metabolic Syndrome as Defined by the ATP III Report. Int. J. Cardiol..

[98] Ridker P.M. (2003). Clinical Application of C-Reactive Protein for Cardiovascular Disease Detection and Prevention. Circulation.

[99] Chu W.-M. (2013). Tumor Necrosis Factor. Cancer Lett..

[100] Navarro J.F., Mora C. (2006). Diabetes, Inflammation, Proinflammatory Cytokines, and Diabetic Nephropathy. Sci. World J..

[101] Lee J.K. (2011). Anti-Inflammatory Effects of Eriodictyol in Lipopolysaccharidestimulated Raw 264.7 Murine Macrophages. Arch. Pharm. Res..

[102] Gamo K., Miyachi H., Nakamura K., Matsuura N. (2014). Hesperetin Glucuronides Induce Adipocyte Differentiation via Activation and Expression of Peroxisome Proliferator-Activated Receptor-γ. Biosci. Biotechnol. Biochem..

[103] Francis G.A., Annicotte J.S., Auwerx J. (2003). PPAR-α Effects on the Heart and Other Vascular Tissues. Am. J. Physiol. Heart. Circ. Physiol..

[104] Rufino A.T., Costa V.M., Carvalho F., Fernandes E. (2021). Flavonoids as Antiobesity Agents: A Review. Med. Res. Rev..

[105] Rohde J., Jacobsen C., Kromann-Andersen H. (2011). Toxic Hepatitis Triggered by Green Tea. Ugeskr. Laeger.

[106] Teschke R., Xuan T.D. (2019). Suspected Herb Induced Liver Injury by Green Tea Extracts: Critical Review and Case Analysis Applying RUCAM for Causality Assessment. Jpn. J. Gastroenterol. Hepatol..

